# Gas-to-Particle Partitioning of Cyclohexene- and α-Pinene-Derived Highly Oxygenated Dimers Evaluated Using COSMO*therm*

**DOI:** 10.1021/acs.jpca.0c11328

**Published:** 2021-04-22

**Authors:** Noora Hyttinen, Matthieu Wolf, Matti P. Rissanen, Mikael Ehn, Otso Peräkylä, Theo Kurtén, Nønne L. Prisle

**Affiliations:** † Nano and Molecular Systems Research Unit, University of Oulu, 90014 Oulu, Finland; ‡ Department of Applied Physics, University of Eastern Finland, 70211 Kuopio, Finland; § Department of Chemistry and Institute for Atmospheric and Earth System Research (INAR), University of Helsinki, 00014 Helsinki, Finland; ∥ Aerosol Physics Laboratory, Physics Unit, Tampere University, 33720 Tampere, Finland; ⊥ Institute for Atmospheric and Earth System Research (INAR)/Physics, University of Helsinki, 00014 Helsinki, Finland; # Center for Atmospheric Research, University of Oulu, 90014 Oulu, Finland

## Abstract

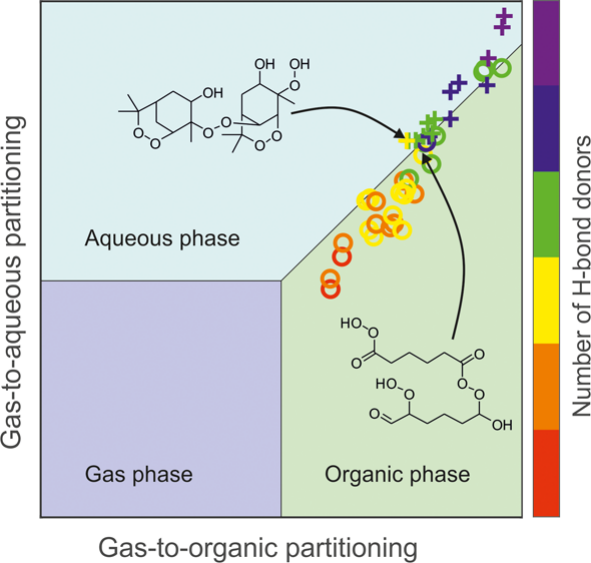

Oxidized organic compounds are expected to contribute to secondary organic aerosol (SOA) if they have sufficiently low volatilities. We estimated saturation vapor pressures and activity coefficients (at infinite dilution in water and a model water-insoluble organic phase) of cyclohexene- and α-pinene-derived accretion products, “dimers”, using the COSMO*therm*19 program. We found that these two property estimates correlate with the number of hydrogen bond-donating functional groups and oxygen atoms in the compound. In contrast, when the number of H-bond donors is fixed, no clear differences are seen either between functional group types (e.g., OH or OOH as H-bond donors) or the formation mechanisms (e.g., gas-phase radical recombination vs liquid-phase closed-shell esterification). For the cyclohexene-derived dimers studied here, COSMO*therm*19 predicts lower vapor pressures than the SIMPOL.1 group-contribution method in contrast to previous COSMO*therm* estimates using older parameterizations and nonsystematic conformer sampling. The studied dimers can be classified as low, extremely low, or ultra-low-volatility organic compounds based on their estimated saturation mass concentrations. In the presence of aqueous and organic aerosol particles, all of the studied dimers are likely to partition into the particle phase and thereby contribute to SOA formation.

## Introduction

Aerosol particles affect the climate through cloud formation and by absorbing and scattering sunlight. Atmospheric organic aerosol is conceptually divided into primary (emitted to the atmosphere as particles, such as pollen) and secondary organic aerosol (SOA; formed in the atmosphere by condensation of vapor). The formation of SOA has been studied extensively in both laboratory and ambient measurements, as well as with theoretical models.^1−7^ The current knowledge suggests that organic compounds with low volatilities are able to condense on existing particles, and in very clean environments and in the laboratory setting, organic compounds have been observed to nucleate without participation of anthropogenic inorganic pollutants, such as sulfuric acid.^8,9^

In the atmosphere, organic compounds with low volatilities are formed when volatile organic compounds (VOCs) react with atmospheric oxidants, such as OH and O_3_. After the initial reaction with an oxidant, molecular oxygen can add to a carbon radical center of the molecule, creating a peroxy radical (RO_2_). In environments under sufficiently low HO_2_ and NO_*x*_ concentrations, the lifetime of the peroxy radical can be sufficiently long for an intramolecular H-shift reaction to take place,^10,11^ which can create a new carbon radical center where another O_2_ then adds. This process can rapidly increase the oxygen content of the molecule. By analogy to other fields in chemistry, this process has been termed autoxidation, and some of the products of such a reaction are highly oxygenated organic molecules (HOMs).^12,13^ Generally, given that the compound does not fragment, the addition of oxygen decreases the volatility of a compound.^14^

Donahue et al.^15^ divided organic compounds into five different volatility groups based on their gas-phase saturation mass concentrations (C*), and recently, a sixth group was added to account for the lowest volatility compounds^16^ (approximate limits for saturation vapor pressures, *p*_sat_)

- VOC with C* > 3 × 10^6^ μg m^–3^ (*p*_sat_ > 10 Pa)
- intermediate volatility organic compounds with 300 < C* < 3 × 10^6^ μg m^–3^ (10^–3^ < *p*_sat_ < 10 Pa)
- semi-volatile organic compounds with 0.3 < C* < 300 μg m^–3^ (10^–6^ < *p*_sat_ < 10^–3^ Pa)
- low-volatility organic compounds (LVOCs) with 3 × 10^–4^ < C* < 0.3 μg m^–3^ (10^–9^ < *p*_sat_ < 10^–6^ Pa)
- extremely low volatility organic compounds (ELVOCs) with 3 × 10^–9^ < C* < 3 × 10^–4^ μg m^–3^ (10^–14^ < *p*_sat_ < 10^–9^ Pa)
- ultra-low-volatility organic compounds (ULVOCs) with C* < 3 × 10^–9^ μg m^–3^ (*p*_sat_ < 10^–14^ Pa)

Volatilities of oxidized organic compounds are often estimated from the carbon oxidation state,^17^ O/C ratio, or chemical structure using group-contribution methods.^18,19^ Compounds with the lowest volatilities (presumably ULVOCs) can nucleate to form new aerosol particles, while the other groups, especially of the lower volatilities, are able to contribute to the growth of existing particles.^9,12,13,16,20^

Rissanen et al. showed experimentally that ozone-initiated oxidation of cyclohexene (C_6_H_10_) leads to the formation of accretion products (often denoted as “dimers”^13^) with 12 carbon atoms and up to 15 oxygen atoms.^10^ Additionally, compounds containing 20 carbon atoms have been detected in many monoterpene (C_10_H_16_) oxidation experiments.^21−24^ Ehn et al.^12^ proposed formation of dimers by a bimolecular radical–radical recombination reaction
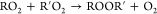
1

This mechanism has previously been thought to occur solely in the condensed phase^25^ but has recently been demonstrated to be feasible also in the gas phase.^26−28^ Another possible formation pathway of dimers is the reaction between two closed-shell reactants, where either a hydroperoxide (ROOH) or an alcohol (ROH) reacts with an aldehyde (R=O) to form a peroxyhemiacetal or hemiacetal, respectively^29^
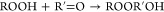
2
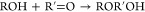
3

These reactions are likely only occurring in the condensed phase because the reactions have high activation energies in the gas phase.^30^ Recently, Kahnt et al.^31^ and Iyer et al.^32^ proposed a formation of a trioxide adduct from a peroxy radical and an alkoxy radical (RO) as a source of atmospheric dimers that have been detected in ambient and chamber measurements in high-loading conditions
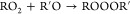
4

All of these reaction pathways can form dimers without loss of carbon atoms from the original monomer reactants and are thus able to form dimers that contain twice the number of carbon atoms compared to the reactant VOC.

Due to the high molecular mass and oxygen content, HOM dimers are presumed to have low volatilities and to contribute to particle formation and growth in the atmosphere.^16^ However, the exact structures of these compounds are still unknown.^13^ Volatilities of individual atmospherically relevant dimers have not been studied experimentally because most atmospherically relevant HOMs are difficult to synthesize in laboratory conditions. In addition, saturation vapor pressures are very difficult to measure accurately.^33^ Experimental methods to measure accurate saturation vapor pressures and activity coefficients of multifunctional compounds, as well as determining the exact chemical structures of multifunctional compounds in trace gas concentrations are not likely to become available in the near future.^33^ Computational methods are therefore crucial in determining properties that are needed for atmospheric models to predict the formation of SOA. The COnductor-like Screening MOdel for Real Solvents (COSMO-RS,^34−36^ implemented in the COSMO*therm* program^37^) has recently been used in various studies to estimate gas-to-particle partitioning of atmospherically relevant compounds.^38−41^ Kurtén et al.^42^ and Hyttinen and Prisle^43^ found that nonsystematic conformer sampling often used in COSMO-RS calculations may lead to large errors in property calculations of multifunctional compounds, while selecting the most energetically favorable conformers with a restricted number of intramolecular H-bonds improves the accuracy of the calculations.^42,43^

Here, we compute saturation vapor pressures of dimers derived from cyclohexene (C_6_H_10_, an anthropogenic pollutant and a model compound for more complex monoterpenes) and α-pinene (C_10_H_16_, a biogenic monoterpene) oxidation reactions, as well as their activity coefficients at infinite dilution in water and water-insoluble organic matter (WIOM). These two properties can be used to estimate gas-to-particle partitioning of atmospherically relevant compounds. The purpose of this work is to evaluate the range of volatilities and activity coefficients of possible accretion products from cyclohexene and α-pinene oxidation in water and a water-insoluble organic and determine the effect of different formation mechanisms, functional groups, and number of oxygen and carbon atoms on volatility and partitioning. Products of the different dimer formation reactions were selected to investigate whether their predicted thermodynamic properties differ significantly. Ozone-initiated autoxidation of cyclohexene and OH-initiated autoxidation of α-pinene have been studied both experimentally and computationally,^10,26,44−50^ and many monomer intermediates have been identified in systematic computational studies of these autoxidation mechanisms.^10,49^ Since exact chemical structures are needed for COSMO*therm* calculations, we have selected these two reaction systems for our investigation, and omitted, for example, the related α-pinene + O_3_ system, as the exact mechanism for autoxidation in that system (forming the highly oxidized monomers) has not been suggested until very recently.^51^

## Methods

### Thermodynamic Properties

Saturation vapor pressures and activity coefficients of the studied dimers were estimated using COSMO-RS theory implemented in the COSMO*therm* program, version 19 (COSMO*therm*19^37^). It has been shown that the COSMO-RS theory gives reasonable saturation vapor pressure estimates, albeit with a tendency to overestimate experimental values for systems studied so far.^52,53^ Schröder et al. compared experimental and COSMO*therm*14-estimated saturation vapor pressures and found the largest overestimation in a compound containing 3 H-bond donors, while COSMO*therm*-estimated saturation vapor pressures of other compounds containing up to 2 H-bond donors agreed better with experiments.^54^ With recent improvements in conformer selection for COSMO*therm* calculations, used also in this study, COSMO*therm* estimates agree well with the limited experimental saturation vapor pressures and activity coefficient data available for atmospherically relevant multifunctional compounds.^42,43^ Below, we shortly describe how COSMO*therm* calculates these thermodynamic properties, and a full description of the COSMO*therm* program can be found in the COSMO*therm* User’s Manual.^55^

At infinite dilution in phase α, the activity coefficient (γ_i_) of compound i can be expressed as
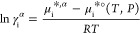
5where μ_i_^*,α^ and μ_i_^*°^(*T*,*P*) are the pseudochemical potentials^56^ of compound i at infinite dilution in α and in its reference state (here chosen as the pure compound i), respectively, *P* is the pressure (10^5^ Pa), *T* is the temperature (298.15 K in all of the calculations of this study), and *R* is the gas constant. Saturation vapor pressure (*p*_sat,i_) of a compound is estimated using the free energy difference of the compound in the pure condensed phase (*G*_i_^(l)^) and in the gas phase (*G*_i_^(g)^)
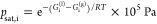
6

We additionally estimate *p*_sat_ values of the studied cyclohexene-derived dimers using the SIMPOL.1 group-contribution method^57^ to compare with COSMO*therm* estimates. In SIMPOL.1, *p*_sat_ is estimated as a sum of contributions from different functional groups
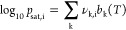
7where *b*_k_ is the temperature-dependent group-contribution term of functional group type k, ν_k_ is the number of functional groups of type k in the compound, and the contributions are summed over all functional groups k. The group-contribution terms of different functional groups are taken from Pankow and Asher.^57^ There are also other well-known group-contribution methods developed to estimate *p*_sat_, such as EVAPORATION^58^ and a method by Nannoolal et al.^59^ However, the Nannoolal et al.^59^ method does not include a descriptor for the peroxy acid group, and EVAPORATION does not include a separate descriptor for a peroxide group; instead, oxygen atoms in the carbon chain are counted in the total number of carbon atoms. Compernolle et al.^58^ furthermore caution that the EVAPORATION method may perform poorly in predicting *p*_sat_ for compounds containing peroxide groups.

Using the COSMO*therm*-estimated activity coefficients at infinite dilution and saturation vapor pressures, we can derive Henry’s law solubilities and saturation mass concentrations of the dimers. The gas-phase saturation mass concentration (*C*_i_^*^) is related to the *p*_sat_ and γ of compound i via
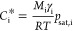
8where *M*_i_ is the molar mass of the compound i. The activity coefficient (γ_i_) is assumed to be unity (ideal solution), which may be reasonable for the equilibrium of an organic compound with respect to an organic phase of similar composition but can lead to large errors for dilute aqueous solutions.^60^ Considering phase α as a solvent, Henry's law solubility can be estimated from the *p*_sat_ and γ^α^
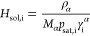
9where ρ_α_ is the density of the pure solvent. Here, the solubility of compound i in the solvent (*x*_sol,i_) is assumed to be very small such that it can be approximated as the inverse of the activity coefficient at infinite dilution
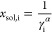
10

Partitioning of a compound i between the gas phase and the organic or aqueous phases can be compared by converting C* and *H*_sol_ into partitioning coefficients. The gas-to-organic partitioning coefficient (*K*_org/G,i_) of compound i can be estimated from
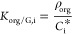
11where we have assumed an organic density of ρ_org_ = 10^12^ μg m^–3^ for simplicity.^40,61^ Here, the organic phase is assumed to be ideal with respect to the partitioning organic compounds, that is, a mixture or organic compounds similar to the dimers. Henry’s law solubility can similarly be used to estimate gas-to-aqueous partitioning coefficient (*K*_w/G,i_) of the organic compound^38,40^
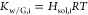
12

Partitioning from one liquid phase to another can be estimated from the activity coefficients of the compound at infinite dilution in each phase and the densities of the two phases. We estimate the partitioning of dimers between water and a WIOM phase. Water-to-WIOM partitioning coefficient of compound i is calculated as
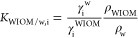
13

As the model WIOM, we use molecule B by Kalberer et al.^62^ (1-(5-(3,5-dimethylphenyl)dihydro-[1,3]dioxolo[4,5-*d*][1,3]dioxol-2-yl)ethan-1-one), which is often used in atmospheric studies as a proxy for the organic particle phase.^38,42,63^ Here, ρ_w_ is the density of water (0.9971 × 10^12^ μg m^–3^), and the density of WIOM (ρ_WIOM_ = 1.2153 × 10^12^ μg m^–3^) was estimated using COSMO*therm*.

### Input File Generation

Conformers of the dimer structures are found using the Spartan program.^64^ A Monte Carlo sampling algorithm is used instead of systematic sampling, due to the high number of torsional degrees of freedom of the dimers. The input files for COSMO*therm* calculations (i.e., cosmo files of each conformer) are generated using the COSMO*conf* program version 4.3,^65^ which uses TURBOMOLE version 7.4.1^66^ for all quantum chemical calculations. In addition to *xyz* coordinates, cosmo-files contain energy information and details of the screening charge surface surrounding the conformer (see Section S1 of the Supporting Information). We use the default COSMO*conf* template (BP_TZVPD_FINE-COSMO.xml)^65^ after omitting the steps involving conformer sampling and removal of conformers based on the number and energy cutoffs in order to keep as many conformers as possible. In this template, the geometries of the initial conformers are optimized at different levels of theory, and conformers are removed after each quantum chemical calculation based on similarities in both geometries (bond length and angle thresholds are 0.5 Å and 20°, respectively) and chemical potentials (with 0.84 kJ mol^–1^ threshold). The final condensed-phase input files are calculated at the BP/def2-TZVPD-FINE//BP/def-TZVP level of theory.

Gas-phase geometries, corresponding to each cosmo geometry, are optimized at the BP/def-TZVP level of theory, and single-point energies are calculated at the BP/def2-TZVPD level of theory. The calculate function of TURBOMOLE is used for the gas-phase calculations using the cosmo files as initial structures. In this way, the condensed-phase conformers correspond to the gas-phase conformers, and the dimer conformers can be divided into groups based on their intramolecular hydrogen bonding, as described by Hyttinen and Prisle^43^ and Kurtén et al.^42^ However, instead of using full and partial intramolecular H-bonds as described in these previous studies, we group the conformers based on the total number of intramolecular H-bonds.

Figures S2–S5 of the Supporting Information show chemical structures of potential dimers studied here. The studied cyclohexene-derived dimers are mainly peroxide products of O_3_-initiated oxidation formed by reactions 1 and 2, representative for gas-phase and condensed-phase peroxide formation, respectively. To investigate the effect of the number of carbon atoms on COSMO*therm*-estimated properties, we included cyclobutene- and cyclopentene-derived dimers similar to two of the cyclohexene-derived dimers (cyclohexene-O8-1 from reaction 1 and cyclohexene-O8-5 from reaction 2). Additionally, we selected one dimer product of reaction 3 (cyclohexene-O10-5) and a trioxide formed in the reaction 4 (cyclohexene-O10-13) to see whether their properties are significantly different than the properties of peroxide dimers. The studied dimers formed in gas-phase reactions contain two fewer hydrogen atoms than those formed from closed-shell monomers. All of the studied α-pinene-derived dimers are products of OH-initiated oxidation formed in reaction 1 and have the same elemental composition. The formation of the studied dimers is explained in more detail in Section S2 of the Supporting Information.

### Conformer Selection

Recently, Kurtén et al. demonstrated the importance of conformer selection in COSMO*therm*-estimated saturation vapor pressures of multifunctional compounds.^42^ With relatively large multifunctional compounds, the selection of conformers in COSMO*therm* calculations becomes important because not all conformers can be included in the calculation due to memory limitations of the program.

**Figure 1:**
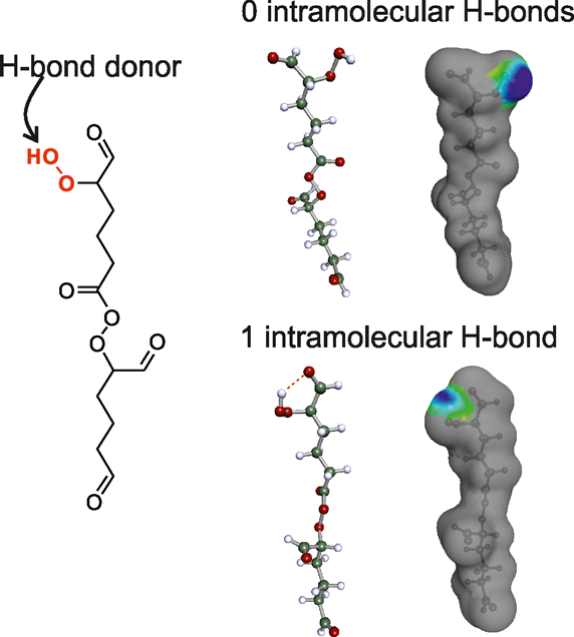
2D structure of the cyclohexene-O8-1 isomer (left hand side) and 3D structures of two conformers containing 0 and 1 intramolecular H-bonds (right hand side). The H-bond donor is highlighted in red. Color coding in the 3D structures: green = C, white = H, red = O, and screening charge surface of the conformer associated with the H-bond donor hydrogen: blue = positive, green = neutral partial charge.

In the context of this study, an H-bond-donating functional group (H-bond donor) is defined by a hydrogen atom that is covalently bound to an oxygen atom. In the studied dimers, all hydroxy (OH), hydroperoxy (OOH), and peroxy acid (C(O)OOH) groups are H-bond donors. These functional groups are able to form intra- and intermolecular H-bonds with hydrogen bond-accepting functional groups. H-bond-accepting functional groups include all oxygen atoms of the molecule, even the ones attached to the H-bond donors. Figure 1 shows, as an example, one of the studied dimers that contains one H-bond donor (OOH) and eight H-bond acceptors.

COSMO*therm* determines whether an H-bond donor of a certain conformer is partially or fully free for intermolecular interactions with surrounding molecules based on the screening charge surface of the conformer. Here, we define an intramolecular H-bond as a H-bond donor that is not able to fully interact with the surrounding mixture (as an H-bond donor) due to intramolecular interactions. From Figure 1, we can see how the conformer that contains an intramolecular H-bond has less positive partial charge (blue surface) around the hydrogen of the H-bond donor than the conformer that contains no intramolecular H-bonds.

Hyttinen and Prisle found that in aqueous solutions, conformers containing fewer intramolecular H-bonds are energetically more favorable (in terms of lower chemical potential) than conformers containing multiple H-bonds.^43^ We tested the effect of intramolecular H-bonds on the favorability of conformers in WIOM and the pure compound and found that conformers containing fewer intramolecular H-bonds are more favorable in both of these solutions (see Section S3 of the Supporting Information). The increased stability in the condensed phase of conformers that contain free H-bond donors is explained by their greater ability to interact with H-bond acceptors of surrounding molecules. For all dimers, the conformer set containing the lowest number of intramolecular H-bonds and at least five conformers in total is used in the COSMO*therm* calculations. For each calculation, the total number of conformers is limited to 40 due to memory restrictions in COSMO*therm*.

## Results

### Saturation Vapor Pressures

**Figure 2:**
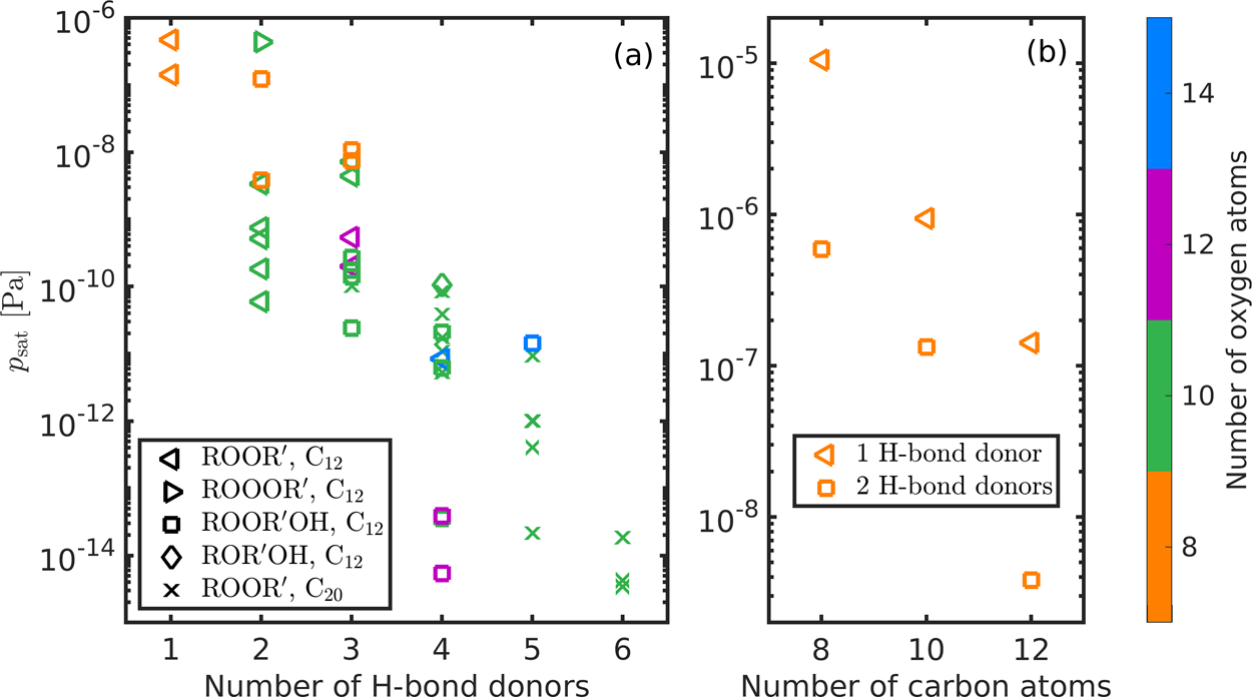
COSMO*therm*-estimated saturation vapor pressures of (a) cyclohexene and α-pinene-derived dimers as a function of the number of H-bond donors and (b) cyclobutene-, cyclopentene-, and cyclohexene-derived dimers as a function of the number of carbon atoms in each molecule. Different markers represent different dimer formation reactions (triangles and crosses for gas-phase, and squares and diamonds for condensed-phase reactions), and the number of oxygen atoms is indicated with different colors. The values are shown in Tables S2 and S3 of the Supporting Information.

The COSMO*therm*-estimated saturation vapor pressures of the studied cyclohexene- and α-pinene-derived dimers are shown in Figure 2a as a function of the number of H-bond donors in each dimer. Figure 2b shows saturation vapor pressures of cyclobutene- and cyclopentene-derived dimers (C_8_ and C_10_, respectively) corresponding to cyclohexene-O8-1 (1 H-bond donor) and cyclohexene-O8-5 (2 H-bond donors) as a function of carbon number. Saturation vapor pressures as a function of the number of free H-bond donors (H-bond donors not forming intramolecular H-bonds) are shown in Figure S6 of the Supporting Information.

There is a clear decrease in *p*_sat_ with the increasing number of H-bond donors. The same effect of H-bond donors on COSMO*therm*-estimated *p*_sat_ has previously been observed in α-pinene-derived organosulfates.^67^ The number of H-bond donors affects condensed-phase interactions more than H-bond acceptors because multifunctional compounds always contain the same number or more H-bond acceptors (all oxygen atoms) than H-bond donors. In addition, decreasing the number of carbon atoms from 12 (cyclohexene-derived dimers) to 8 (cyclobutene-derived dimers) increases the saturation vapor pressure by around 0.5 order of magnitude per carbon atom.

**Figure 3:**
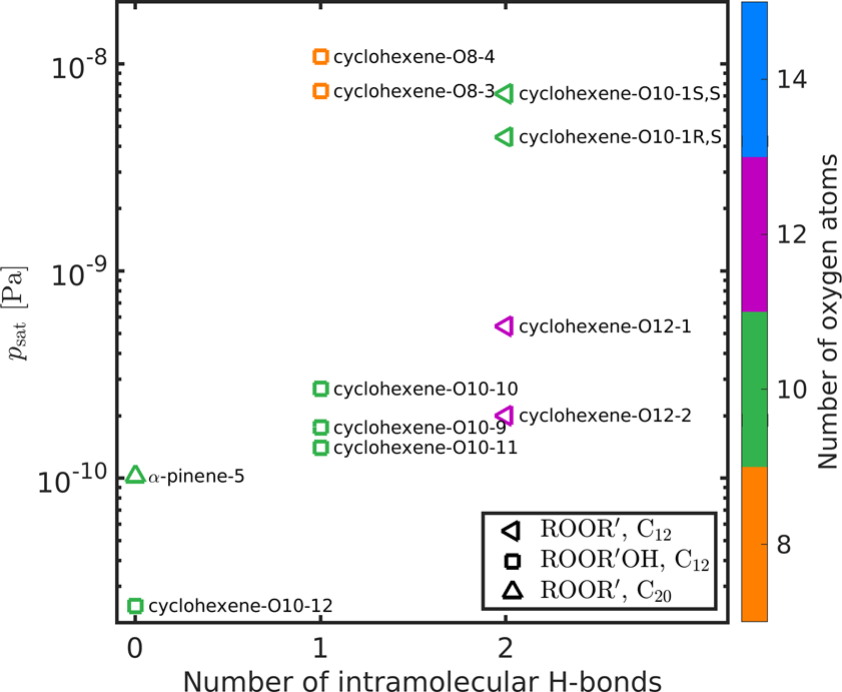
COSMO*therm*-estimated saturation vapor pressures of cyclohexene and α-pinene-derived dimers as a function of the maximum number of intramolecular H-bonds in the conformer set used in the COSMO*therm* calculation (three H-bond donors). Different markers represent different dimer formation reactions (triangles for gas-phase and squares for condensed-phase reactions), and the number of oxygen atoms is shown with different colors. The chemical structures of the compounds are shown in Figures S2–S4 of the Supporting Information.

Figure 3 shows vapor pressures of the studied dimers containing three H-bond donors as a function of the maximum number of intramolecular H-bonds in the conformer set used in the COSMO*therm* calculation because we were not able to find five conformers without any intramolecular H-bond for all the studied dimers. (In COSMO*therm* calculations where conformers contain intramolecular H-bonds, the conformer set may include up to 4 conformers that contain fewer intramolecular H-bond if their COSMO-energies are within the 40 lowest-energy conformers of the used conformer set.) Based on the findings of Kurtén et al.,^42^ the vapor pressures obtained using conformers containing no intramolecular H-bonds are likely closest to the real saturation vapor pressures. Similar figures for two, four, and five H-bond donors are shown in Figures S7–S9 of the Supporting Information.

From Figure 3, we can see how the selection of conformer sets affects the *p*_sat_ estimate in COSMO*therm*, and the dimers with the similar conformer sets can be compared with each other. For example, we see that the cyclohexene-derived dimers that contain 8 oxygen atoms have higher *p*_sat_ than the dimers that contain 10 oxygen atoms when conformers containing the same number of intramolecular H-bonds are used in the COSMO*therm* calculation. Similarly, dimers containing 12 oxygen atoms have lower *p*_sat_ than those containing 10 oxygen atoms. This comparison indicates that the addition of an oxygen atom to a multifunctional compound decreases the *p*_sat_ of a compound by around 0.5–1 order of magnitude when the number of H-bond donors and the number of H-bonds in the COSMO*therm* calculation are kept constant.

There is no clear difference in the COSMO*therm*-estimated *p*_sat_ of the studied dimers formed in different dimerization reactions (different markers in Figures 2 and 3) when the number of H-bond donors does not change. However, the dimers selected for this study formed in gas-phase reactions contain fewer H-bond donors than the dimers with the same number of oxygen atoms formed in condensed-phase reactions. The reason for this is that the “monomers” forming the gas-phase dimers (reaction 1) are peroxy radicals. By definition, these have at least two oxygen atoms each bound to the peroxy groups, which lack H-bond donors. In contrast, the reactions forming the condensed-phase dimers (reactions 2 and 3) involve groups containing H-bond donors. If the closed-shell reactants of the condensed-phase dimerization reactions are formed by, for example, RO_2_ + HO_2_ termination reactions (which increase the number of H-bond donor groups) of the corresponding gas-phase peroxy radicals, then, for a given number of oxygen atoms, the condensed-phase dimers will inevitably have more H-bond donors than the gas-phase dimers.

Comparing the cyclohexene- and α-pinene-derived dimers that have the same number of H-bond donors and oxygen atoms and that were calculated using conformers containing the same number of intramolecular H-bonds, we see that the α-pinene-derived dimers have higher *p*_sat_ than the cyclohexene-derived dimers (see Figures 3 and S8 of the Supporting Information). This result is surprising, as larger molecules (all else being equal) have lower *p*_sat_ (see Figure 2b). For example, COSMO*therm* predicts that each carbon atom lowers *p*_sat_ by around 0.5 order of magnitude, which would lead to C_20_ compounds having *p*_sat_ about 4 orders of magnitude lower than the equivalent C_12_ compounds. The surprising prediction is likely related to the ring structures in the α-pinene-derived dimers. For example, α-pinene-1 and α-pinene-3 both have two six-membered rings and an additional four-membered carbon ring. The saturation vapor pressures of these two dimers are higher relative to other α-pinene-derived dimers that contain the same number of H-bond donors and intramolecular H-bonds but no four-membered carbon rings. This indicates that ring structures in HOMs lead to higher saturation vapor pressures, likely due to reduced intermolecular interaction in the condensed phase. Since the change in the number of ring structures in the dimer also changes the number of double bonds, when the degree of unsaturation is kept constant, it is impossible to calculate the exact effect of the ring structures on saturation vapor pressure. However, based on the molecules studied here, the effect of ring structures on saturation vapor pressure is expected to be smaller than the effect of the number of H-bond donors. The higher vapor pressure of cyclic, compared to linear, molecules has also been seen experimentally in compounds, such as linear^33^ and cyclic^68^ dicarboxylic acids, as well as in polydimethylsiloxanes.^69^ The effect of some structural differences, including ring structures, on saturation vapor pressure estimates is discussed in more detail in Section S1 of the Supporting Information.

Kurtén et al.^39^ estimated *p*_sat_ of two α-pinene-derived dimers containing 10 oxygen atoms using COSMO*therm*15 (our test calculations indicate that COSMO*therm*-estimated *p*_sat_ using this older parameterization can be more than 1 order of magnitude higher than using the new parametrization due to improvements in the H-bonding parametrization; see Section S4 of the Supporting Information for a comparison). Both of these isomers are proposed structures of O_3_-initiated oxidation of α-pinene, and they contain one and two H-bond donors. Saturation vapor pressures of these two structures, computed at the BP/TZVP level of theory using multiple conformers, were 1.6 × 10^–7^ and 9.0 × 10^–10^ Pa (2.8 × 10^–2^ and 1.6 × 10^–4^ μg m^–3^), respectively. This is in agreement with the present *p*_sat_ estimates: compared to the α-pinene-derived dimers studied here, the oxygenated α-pinene-derived dimers studied by Kurtén et al. contain a relatively high number of carbonyl groups and fewer H-bond donors. Although Kurtén et al. did not omit conformers containing intramolecular H-bonds from their COSMO*therm* calculations, the effect of intramolecular H-bonds on vapor pressure estimates is small due to the low number of H-bond donors (see Figure S11 of the Supporting Information).

### Comparison with SIMPOL.1-Estimated Saturation Vapor Pressures

**Figure 4:**
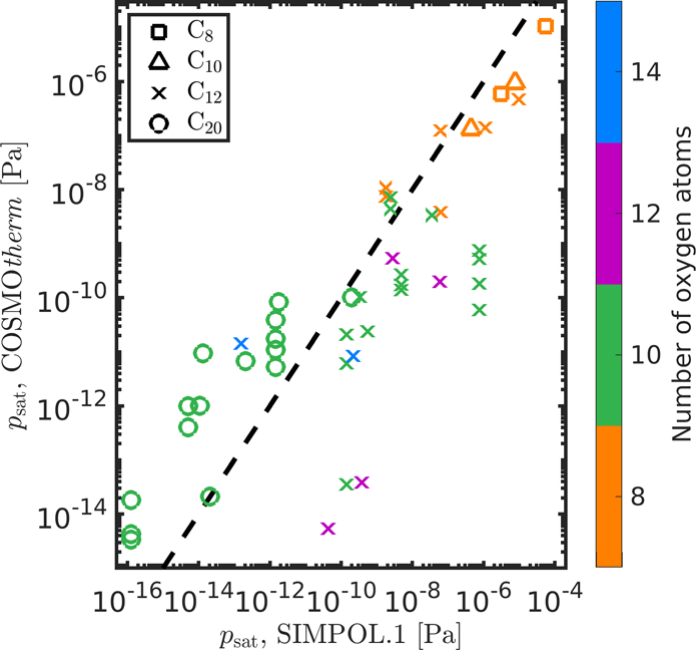
Comparison between COSMO*therm*- and SIMPOL.1-estimated saturation vapor pressures of the studied dimers at 298.15 K. The number of oxygen atoms is shown with different colors. The dashed line shows the 1:1 relation between the methods. The values are shown in Tables S2 and S3 of the Supporting Information.

In Figure 4, we compare COSMO*therm*-estimated *p*_sat_ values of the studied dimers with those calculated using the SIMPOL.1 group-contribution method.^57^ We see that COSMO*therm* predicts a higher *p*_sat_ than SIMPOL.1 for most of the studied α-pinene-derived dimers by up to 3 orders of magnitude. For the studied cyclobutene-, cyclopentene-, and cyclohexene-derived dimers, COSMO*therm* predicts up to 4 orders of magnitude lower *p*_sat_ than SIMPOL.1. This is opposite to what was found in a previous study, where SIMPOL.1 predicted lower *p*_sat_ than COSMO*therm* for mostly monomer HOMs formed in the ozonolysis of α-pinene.^39^ However, in their study, Kurtén et al.^39^ used fewer conformers in the COSMO*therm* calculations and the conformers were not selected based on their intramolecular H-bonding, and instead, conformers were selected to contain different H-bonding patterns. For compounds containing a high number (≥2) of H-bond donors, this may have led to higher *p*_sat_ estimates than using conformers containing no intramolecular H-bonds and a more comprehensive conformer selection.^42^ We therefore recommend the use of a more extensive conformer search than what is available in COSMO*conf*, as well as the newest version of COSMO*therm* in calculations of multifunctional compounds.

Öström et al. used SIMPOL.1- and COSMO*therm*-estimated *p*_sat_ of HOMs to model particle formation at the Pallas Atmosphere-Ecosystem Supersite in Northern Finland.^70^ They found a better agreement between measured and modeled particle numbers using the SIMPOL.1 estimates than higher *p*_sat_ estimated using COSMO*therm*. In their study, the difference between COSMO*therm* and SIMPOL.1 was estimated using COSMO*therm* estimates of predominantly monomer HOMs, using fewer conformers, most of them containing intramolecular H-bonds.^39^ As has been shown previously by Kurtén et al.,^42^ including conformers that contain intramolecular H-bonds generally increases the *p*_sat_ estimates. This also helps explain the previous observations of COSMO*therm* overestimating SIMPOL.1-estimated *p*_sat_ of multifunctional compounds.

Above, we noticed a 0.5–1 order of magnitude decrease in *p*_sat_ with the addition of one oxygen atom to a compound, keeping the number of H-bond donors and intramolecular H-bonds constant. While COSMO*therm*-estimated *p*_sat_ values do not seem to strongly depend on the functional group types of the compound, if the number of H-bond donors is unchanged, different functional groups have significantly different effects on SIMPOL.1-estimated vapor pressures. In SIMPOL.1, the effect of increasing the number of oxygen atoms on *p*_sat_ (while keeping the number of H-bond donors constant) depends on the functional groups. For example, if an acyl-type oxygen is added to a compound, the decrease in SIMPOL.1-estimated vapor pressure is between 0 and 1.3 orders of magnitude. Combining functional groups present in the studied cyclohexene-derived dimers in such a way that they contain three oxygen atoms and one H-bond donor, SIMPOL.1-estimated *p*_sat_ values vary 2.4 orders of magnitude from a combination of one hydroxy and two aldehyde groups (lowest *p*_sat_) to one peroxy acid (highest *p*_sat_). This variation is similar to what is seen among the COSMO*therm*-estimated *p*_sat_ values of dimers containing the same number of oxygen atoms, H-bond donors, and intramolecular H-bonds. In addition, the 0.5 order of magnitude decrease in COSMO*therm*-estimated *p*_sat_ from the addition of each CH_2_ group is similar to the decrease in SIMPOL.1-estimated *p*_sat_ (0.42 order of magnitude for each carbon atom).

### Activity Coefficients

**Figure 5:**
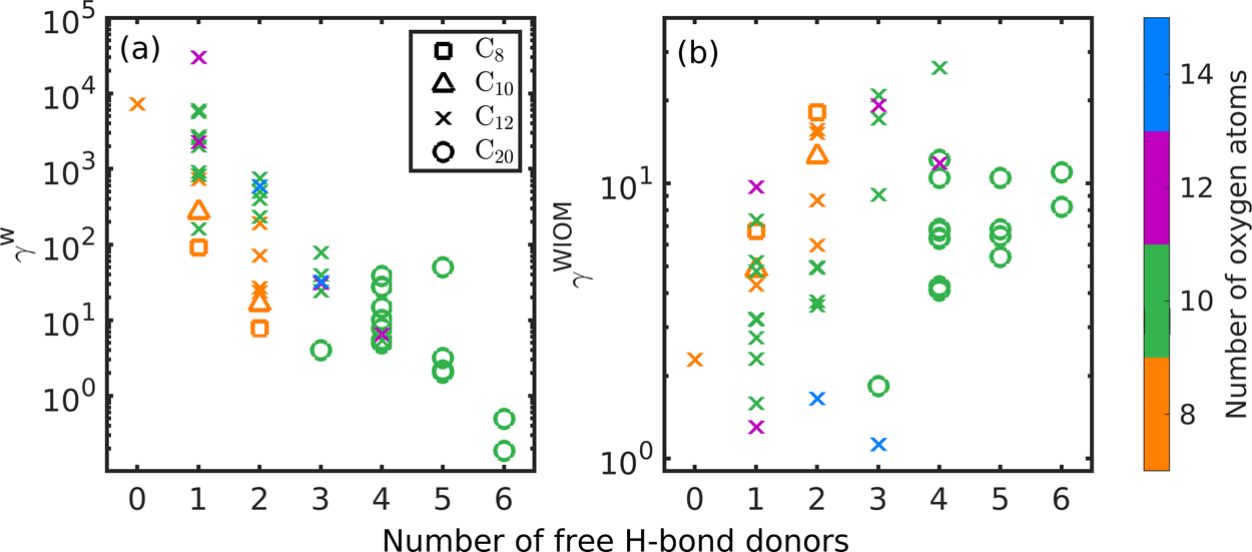
COSMO*therm*-estimated activity coefficients of the studied dimers at infinite dilution in (a) water and (b) WIOM as a function of the number of free H-bond donors. Different markers represent different conformer sets used in COSMO*therm* calculations, and the number of oxygen atoms is shown with different colors. The values are shown in Tables S2 and S3 of the Supporting Information.

Activity coefficients of the studied dimers at infinite dilution in water (γ^w^) and WIOM (γ^WIOM^) are shown in Figure 5 as a function of the free H-bond donors in conformers used in the calculation. Similar to the saturation vapor pressures, there is a decreasing trend in aqueous activity coefficients (γ^w^) with an increasing number of H-bond donors in the dimer (see Figure 5a). This indicates that, as expected, free H-bond donors promote mixing of the organic compound in water, and compounds containing fewer H-bond donors are increasingly hydrophobic. The correlation of aqueous activity coefficients is better with free H-bond donors, as compared to the total number of H-bond donors in the compound. The activity coefficients of the studied dimers as a function of the total number of H-bond donors are shown in Figure S12 of the Supporting Information.

Figure S13 of the Supporting Information shows aqueous activity coefficients of the studied dimers as a function of intramolecular H-bonds in the conformer sets used in the COSMO*therm* calculations. We see that the values calculated using conformers that contain fewer intramolecular H-bonds are lower than those calculated using conformers that contain more intramolecular H-bonds. In addition, Figure S14 of the Supporting Information shows the aqueous activity coefficients of selected cyclohexene-derived dimers calculated using different conformer sets in COSMO*therm*. COSMO*therm* estimates lower γ^w^ for the same compound using conformers containing fewer intramolecular H-bonds than conformers containing multiple H-bonds. As was previously noted by Hyttinen and Prisle, this effect of omitting conformers containing intramolecular H-bonds on aqueous activity coefficients is caused by increased interaction between water and the H-bond donors of the HOM.^43^

In contrast to the saturation vapor pressures, the γ^w^ increases as the number of oxygen atoms increases if the number of H-bond donors is kept constant. This indicates that for these compounds, adding oxygen atoms (negative partial charge) without increasing the number of H-bond donors (positive partial charge) reduces the ability of the compound to mix with water, as the studied dimers have relatively high negative charge densities, while water has equal negative and positive charge densities. However, increasing the number of carbon atoms (α-pinene-derived dimers compared to cyclohexene-derived dimers) seems to have very little, if any, impact on the mixing of dimers with water. It is possible that the types of functional groups in the studied α-pinene-derived dimers compared to the cyclohexene-derived dimers have a large enough effect on activity coefficients to counter the effect of the additional eight carbon atoms in the α-pinene-derived dimers compared to cyclohexene-derived dimers.

COSMO*therm*-estimated activity coefficients in WIOM increase slightly with the addition of H-bond donors. Overall, the activity coefficients of the different dimers at infinite dilution in WIOM are very similar and also quite close to unity. Saturation vapor pressure alone can therefore describe gas-to-particle partitioning of organic aerosol but not aqueous aerosol. In addition, the effect of intramolecular H-bonds in COSMO*therm* calculations is not as significant as in aqueous solutions because the change in energies of different conformers is similar in the pure dimer and WIOM (see Section S3 and Figure S15 of the Supporting Information). Most of the large cyclohexene-O12 and cyclohexene-O14 dimer conformers contain multiple intramolecular H-bonds, leading to less accurate activity coefficient estimates.

### Gas-to-Particle Partitioning

We estimated saturation mass concentrations, Henry’s law solubilities, and partitioning coefficients using the COSMO*therm*-estimated *p*_sat_ and γ (see Tables 1 and 2). These properties are commonly used in atmospheric modeling to describe the gas-to-particle partitioning of SOA constituents. The C* values were calculated assuming ideal solutions (γ = 1), which may be appropriate for organic compounds condensing into a phase comprising similar compounds. For the calculation of gas-to-organic partitioning coefficients, the density of the organic phase is assumed to be 10^12^ μg m^–3^.

**Table 1:** COSMO*therm*-Estimated Cyclohexene-Derived Dimer Saturation Mass Concentration (C* in μg m^–3^) in Ideal (γ = 1) Solution, Henry’s Law Solubility in Water (*H*_sol_ in mol m^–3^ Pa^–1^), and Gas-to-Organic (*K*_org/G_), Gas-to-Aqueous (*K*_w/G_), and Aqueous-to-WIOM (*K*_WIOM/w_) Partitioning Coefficients at 298.15 K^a^

	C*	*H*_sol_	*K*_org/G_	*K*_w/G_	*K*_WIOM/w_	# H-bonds
cyclohexene-O8-1	1.66 × 10^–2^	5.32 × 10^8^	6.03 × 10^13^	1.32 × 10^12^	2.09 × 10^2^	0/1
cyclohexene-O8-2	5.47 × 10^–2^	1.65 × 10^7^	1.83 × 10^13^	4.09 × 10^10^	3.81 × 10^3^	1/1
cyclohexene-O8-3	8.73 × 10^–4^	2.77 × 10^11^	1.15 × 10^15^	6.87 × 10^14^	2.17	1/3
cyclohexene-O8-4	1.28 × 10^–3^	2.17 × 10^11^	7.82 × 10^14^	5.37 × 10^14^	1.83	1/3
cyclohexene-O8-5	4.52 × 10^–4^	2.02 × 10^11^	2.21 × 10^15^	5.00 × 10^14^	1.00 × 10^1^	0/2
cyclohexene-O8-6	1.45 × 10^–2^	2.34 × 10^9^	6.88 × 10^13^	5.81 × 10^12^	3.91 × 10^1^	0/2
cyclohexene-O10-1*R*,*S*	5.77 × 10^–4^	4.61 × 10^9^	1.73 × 10^15^	1.14 × 10^13^	1.03 × 10^3^	2/3
cyclohexene-O10-1*S*,*S*	9.36 × 10^–4^	9.36 × 10^9^	1.07 × 10^15^	2.32 × 10^13^	1.93 × 10^2^	2/3
cyclohexene-O10-2*R*,*S*	7.84 × 10^–6^	4.55 × 10^11^	1.28 × 10^17^	1.13 × 10^15^	7.63 × 10^2^	1/2
cyclohexene-O10-2*S*,*S*	6.76 × 10^–5^	1.88 × 10^10^	1.48 × 10^16^	4.65 × 10^13^	2.50 × 10^3^	1/2
cyclohexene-O10-3*R*,*S*	2.40 × 10^–5^	1.85 × 10^12^	4.17 × 10^16^	4.58 × 10^15^	2.69 × 10^1^	1/2
cyclohexene-O10-3*S*,*S*	9.86 × 10^–5^	1.23 × 10^10^	1.01 × 10^16^	3.04 × 10^13^	3.15 × 10^3^	1/2
cyclohexene-O10-4	4.42 × 10^–4^	1.78 × 10^10^	2.26 × 10^15^	4.42 × 10^13^	2.31 × 10^2^	1/2
cyclohexene-O10-5	1.37 × 10^–5^	2.26 × 10^12^	7.28 × 10^16^	5.60 × 10^15^	7.61 × 10^1^	2/4
cyclohexene-O10-6	8.08 × 10^–7^	2.27 × 10^14^	1.24 × 10^18^	5.62 × 10^17^	2.31	1/4
cyclohexene-O10-7	2.73 × 10^–6^	1.08 × 10^14^	3.66 × 10^17^	2.69 × 10^17^	1.73	1/4
cyclohexene-O10-8	4.67 × 10^–9^	2.87 × 10^17^	2.14 × 10^20^	7.12 × 10^20^	2.50 × 10^–1^	0/4
cyclohexene-O10-9	2.30 × 10^–5^	4.17 × 10^11^	4.35 × 10^16^	1.03 × 10^15^	2.56 × 10^2^	1/3
cyclohexene-O10-10	3.54 × 10^–5^	5.11 × 10^11^	2.83 × 10^16^	1.27 × 10^15^	9.90 × 10^1^	1/3
cyclohexene-O10-11	1.84 × 10^–5^	7.88 × 10^11^	5.44 × 10^16^	1.95 × 10^15^	1.23 × 10^2^	1/3
cyclohexene-O10-12	3.17 × 10^–6^	2.92 × 10^13^	3.16 × 10^17^	7.23 × 10^16^	1.05 × 10^1^	0/3
cyclohexene-O10-13	5.70 × 10^–2^	5.02 × 10^7^	1.76 × 10^13^	1.24 × 10^11^	1.93 × 10^3^	1/2
cyclohexene-O12-1	7.74 × 10^–5^	4.51 × 10^10^	1.29 × 10^16^	1.12 × 10^14^	2.83 × 10^2^	2/3
cyclohexene-O12-2	2.86 × 10^–5^	9.18 × 10^9^	3.49 × 10^16^	2.28 × 10^13^	2.81 × 10^4^	2/3
cyclohexene-O12-3	7.83 × 10^–10^	3.30 × 10^17^	1.28 × 10^21^	8.18 × 10^20^	1.95	1/4
cyclohexene-O12-4	5.59 × 10^–9^	2.17 × 10^17^	1.79 × 10^20^	5.38 × 10^20^	6.77 × 10^–1^	0/4
cyclohexene-O14-1	1.32 × 10^–6^	1.11 × 10^13^	7.60 × 10^17^	2.74 × 10^16^	4.36 × 10^2^	2/4
cyclohexene-O14-2	2.26 × 10^–6^	1.18 × 10^14^	4.43 × 10^17^	2.92 × 10^17^	1.29 × 10^1^	2/5

a Number of intramolecular H-bonds in the conformers used in the COSMO*therm* calculation and the total number of H-bond donors are given in the # H-bonds column (intramolecular H-bonds/H-bond donors).

**Table 2:** COSMO*therm*-Estimated α-Pinene-Derived Dimer Saturation Mass Concentration (C* in μg m^–3^) in Ideal (γ = 1) Solution, Henry’s Law Solubility in Water (*H*_sol_ in mol m^–3^ Pa^–1^), and Gas-to-Organic (*K*_org/G_), Gas-to-Aqueous (*K*_w/G_), and Aqueous-to-WIOM (*K*_WIOM/w_) Partitioning Coefficients at 298.15 K^a^

	C*	*H*_sol_	*K*_org/G_	*K*_w/G_	*K*_WIOM/w_	# H-bonds
α-pinene-1	1.48 × 10^–5^	1.33 × 10^14^	6.76 × 10^16^	3.29 × 10^17^	1.42	0/4
α-pinene-2*R*	6.87 × 10^–6^	1.39 × 10^14^	1.46 × 10^17^	3.45 × 10^17^	1.96	0/4
α-pinene-2*S*	3.06 × 10^–6^	2.15 × 10^14^	3.27 × 10^17^	5.33 × 10^17^	2.63	0/4
α-pinene-3	1.65 × 10^–6^	2.15 × 10^14^	6.05 × 10^17^	5.33 × 10^17^	5.25	1/5
α-pinene-4	1.78 × 10^–7^	1.41 × 10^15^	5.61 × 10^18^	3.49 × 10^18^	7.00	1/5
α-pinene-5	1.79 × 10^–5^	1.35 × 10^14^	5.58 × 10^16^	3.34 × 10^17^	2.66	0/3
α-pinene-6	1.93 × 10^–6^	9.22 × 10^14^	5.19 × 10^17^	2.29 × 10^18^	6.32 × 10^–1^	0/4
α-pinene-7	9.24 × 10^–7^	1.35 × 10^15^	1.08 × 10^18^	3.35 × 10^18^	7.77 × 10^–1^	0/4
α-pinene-8	3.71 × 10^–9^	5.20 × 10^16^	2.70 × 10^20^	1.29 × 10^20^	5.83	0/5
α-pinene-9*R*,*R*	5.97 × 10^–10^	8.73 × 10^19^	1.67 × 10^21^	2.17 × 10^23^	2.76 × 10^–2^	0/6
α-pinene-9*S*,*R*	7.49 × 10^–10^	2.63 × 10^19^	1.34 × 10^21^	6.52 × 10^22^	5.45 × 10^–2^	0/6
α-pinene-9*S*,*S*	3.23 × 10^–9^	9.43 × 10^17^	3.09 × 10^20^	2.34 × 10^21^	5.70 × 10^–1^	1/6
α-pinene-10*R*	1.75 × 10^–7^	2.56 × 10^16^	5.72 × 10^18^	6.36 × 10^19^	4.87 × 10^–1^	0/5
α-pinene-10*S*	7.13 × 10^–8^	6.72 × 10^16^	1.40 × 10^19^	1.67 × 10^20^	3.83 × 10^–1^	0/5
α-pinene-11	1.18 × 10^–6^	2.94 × 10^14^	8.46 × 10^17^	7.29 × 10^17^	8.33	0/4

a Number of intramolecular H-bonds in the conformers used in the COSMO*therm* calculation and the total number of H-bond donors are given in the # H-bonds column (intramolecular H-bonds/H-bond donors).

**Figure 6:**
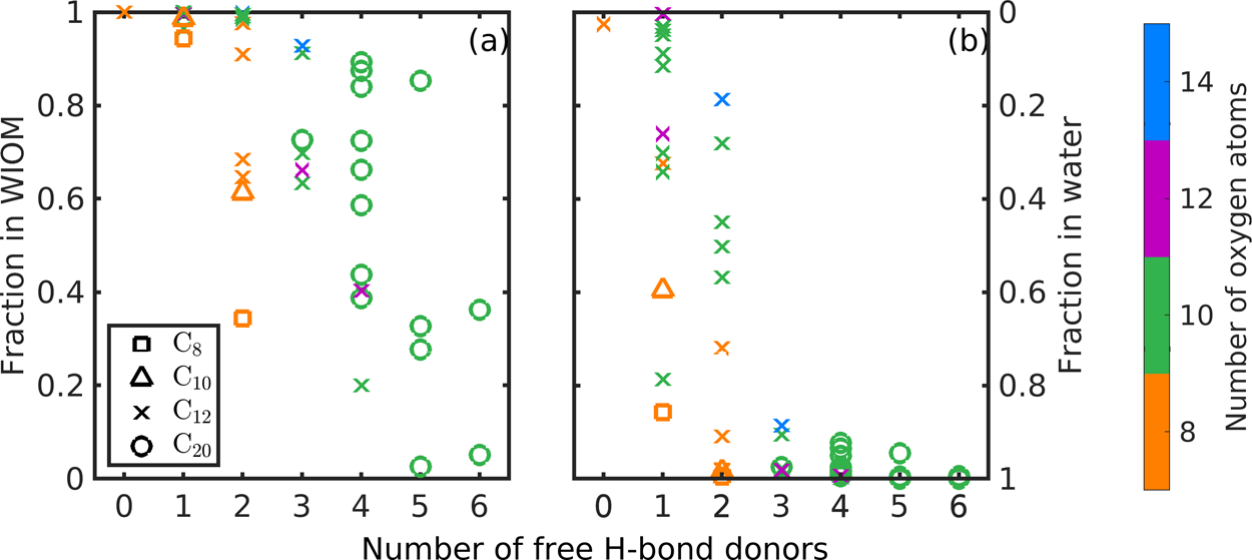
Fractions of dimers in WIOM (left *y*-axis) and water (right *y*-axis) in a system that contains (a) 1:1 WIOM and water and (b) 100 times more water than WIOM. Different markers represent the carbon number of the dimer.

Figure 6 shows how the studied dimers partition to the WIOM and aqueous phases in a system that contains equal volumes of WIOM and water (Figure 6a) and 100 times more water than WIOM (Figure 6b). These systems represent conditions of low and moderate relative humidity, respectively. Additional figures for a cloud scenario (3 × 10^4^ times more water than WIOM^40^) and dry conditions (100 times more WIOM than water) are shown in Figures S16 and S17 of the Supporting Information, respectively. Here, a clear trend is seen in the effect of the chain length (C_8_, C_10_, and C_12_). As can be seen from Figure 5, increasing the number of carbon atoms from 8 to 12 leads to an increase and decrease in activity coefficient at infinite dilution in water and WIOM, respectively. Both of these changes lead to stronger partitioning to the aqueous phase of the dimers with lower carbon numbers, compared to the corresponding cyclohexene-derived dimers.

## Discussion and Conclusions

On the basis of the C* values shown in Tables 1 and 2, all of the studied cyclohexene-derived dimers can be categorized as LVOCs, ELVOCs, or ULVOCs, while all of the studied α-pinene-derived dimers are ELVOCs or ULVOCs. The distinction between ELVOC and ULVOC is crucial, especially in clean environments with few pre-existing aerosol particles, where ULVOCs can nucleate to form new aerosol particles in addition to condensing onto pre-existing particles. In polluted environments, both ELVOCs and ULVOCs likely contribute to the growth of existing particles. There is a general decrease in the volatility with the addition of oxygen and carbon atoms to the compound. The addition of H-bond donors leads to lower volatilities, while intramolecular H-bonds lead to higher volatilities. The effect of number of H-bond donors is more clear in the studied α-pinene-derived dimers, for which we were able to find a sufficient number of conformers without intramolecular H-bonds.

We compared our C* values with those obtained from various group-contribution expressions. For example, Donahue et al.,^18^ Bianchi et al.,^13^ and Peräkylä et al.^71^ derived simple equations for experimentally determined C* using the numbers of carbon, oxygen, and hydrogen atoms as variables. Since these equations only depend on the elemental composition of a compound, identical C* values are predicted for all studied α-pinene-derived dimers with the same elemental composition. The equation by Bianchi et al. gives the best agreement with our C* values for the studied cyclohexene-derived dimers out of the three above mentioned equations (see Figure S18 of the Supporting Information). Peräkylä et al.^71^ derived their equation for HOM monomers from α-pinene + O_3_ experiments, also where OH is formed. Saturation mass concentrations derived using the equation by Peräkylä et al. are much higher than those calculated from COSMO*therm* estimates, and the difference between the two estimates increases with the increasing number of H-bond donors from around 1 order of magnitude for the cyclohexene-derived dimers containing a single H-bond donor to 7 orders of magnitude for the α-pinene-derived dimers that contain 6 H-bond donors. This is likely related to the fact that the HOM compounds from α-pinene + O_3_ oxidation contain fewer H-bond donors than the dimers from α-pinene + OH oxidation studied here and that the relationship was derived primarily for HOM monomers. Using the equations by Donahue et al.^18^ and Peräkylä et al.,^71^ the addition of oxygen atoms has a constant effect on C*: 1.7 or 0.4 orders of magnitude decrease in C* per one additional oxygen atom, respectively. In the equation by Bianchi et al.,^13^ the change in C* depends on both the change in the oxygen number and the number of carbon atoms in the compound. For the studied cyclohexene-derived dimers, the equation by Bianchi et al. predicts 0.3–0.4 order of magnitude decrease in C* with the addition of one oxygen atom. The effect of additional oxygen atoms on C* estimated by Bianchi et al.^13^ and Peräkylä et al.^71^ is therefore close to the COSMO*therm*-estimated 0.5–1 order of magnitude.

Kurtén et al. predicted that highly oxygenated products of α-pinene ozonolysis may not have as low volatilities as was previously thought.^39^ While this may be true especially for HOM monomers, we show here how the estimated volatilities of the studied HOM dimers are highly dependent on the number of H-bond donors, which means that compounds with the same elemental composition can have very different volatilities depending on the chemical structure. The volatility of HOMs therefore depend on the formation reaction through the types of functional groups that are formed. In ozonolysis reactions of endocyclic alkenes, the initial O_3_ addition leads to the formation of two carbonyl groups.^10^ On the contrary, the OH reaction is likely to add more H-bond donors to the product molecule through OH addition to the double bond and hydroperoxide formation subsequent to the H abstraction reaction. Thus, final products that contain the same number of oxygen atoms are likely to have more H-bond donors when the initial oxidant is the OH radical rather than O_3_. However, α-pinene + OH produces HOM that contains fewer oxygen atoms than α-pinene + O_3_.^48,51^ Based on C* values estimated here, at least 10 oxygen atoms and 2 H-bond donors are needed for the HOM dimer to be an ELVOC, while less oxygenated dimers are more likely to be LVOCs. This will significantly assist in inferring volatilities from mass spectrometric measurements, as the number of H-bond donors can be measured with relative ease by mixing the samples with deuterated water.^12^

Krieger et al. found that COSMO*therm* overestimates experimental saturation vapor pressures of polyethylene glycol by a factor of 3–40.^53^ Validation of COSMO*therm*-estimated saturation vapor pressure of isoprene-derived C_5_H_12_O_6_ and C_5_H_10_O_6_ with experimental values showed a maximum error of a factor of 5 using conformer sets with a limited number of intramolecular H-bonds in the COSMO*therm* calculations.^42^ In addition, each intramolecular H-bond in the used conformer set is expected to lead to a factor of 5 overestimation in COSMO*therm*-estimated saturation vapor pressures.^42^ We therefore estimate the potential uncertainty in *p*_sat_, C*, *H*_sol_, *K*_org/G_, and *K*_w/G_ to be a factor of 5 in cases where conformers containing no intramolecular H-bonds were used in the COSMO*therm* calculation, with an additional factor of 5 uncertainty for each intramolecular H-bond. Hyttinen and Prisle found good agreement between experimental and COSMO*therm*-estimated activity coefficients of multifunctional carboxylic acids.^43^ However, there are no experimental data available for multifunctional compounds at infinite dilution with respect to the pure compound. Without experimental values, we are not able to estimate uncertainties in the activity coefficients at infinite dilution. Henry’s law solubility is commonly used to model the partitioning of organic molecules in aqueous SOA particles as it takes the nonideality of the (aqueous) aerosol into account. Henry’s law solubilities in water are estimated using both *p*_sat_ and γ^w^. The effect of intramolecular H-bonds in the COSMO*therm* calculations is therefore multiplied, leading to a larger effect of conformer selection in COSMO*therm* calculations.

Wania et al.^38^ modeled the phase distribution of organic compounds in typical atmospheric conditions (10 μg of both organic and water per 1 m^3^ of air). According to their model, 1, 10, 50, 90, and 99% of the organic compound is in the particle phase when its gas-to-particle partitioning coefficients are 10^9^, 10^10^, 10^11^, 10^12^, and 10^13^, respectively. All of the studied dimers have gas-to-organic partitioning coefficients above 10^13^, indicating that, in typical atmospheric conditions of the model, in equilibrium, >99% of the dimers would be either in the organic or aqueous particle phase. However, we note that especially for the dimers formed by gas-phase reactions, this equilibrium may not be reached in realistic atmospheric conditions. With few exceptions, the studied cyclohexene-derived dimers, having high aqueous activity coefficients (γ^w^ > 1), are more likely to partition to the organic than aqueous phase (*K*_org/G_/*K*_w/G_ > 1). On the contrary, the studied α-pinene-derived dimers with activity coefficients closer to unity in water, have higher partitioning to the aqueous than organic phase (*K*_org/G_/*K*_w/G_ < 1). This explains the observation by Peräkylä et al., that for some α-pinene + O_3_ HOM compounds, gas-to-particle partitioning is stronger in humid than in dry conditions.^71^

Figure 6 shows how a higher number of H-bond donors leads to a larger fraction of the dimer partitioning to the aqueous phase, as opposed to the WIOM phase. In a system that contains equal amounts of WIOM and water (Figure 6a), only some of the studied dimers with six H-bond donors are predominantly found in the aqueous phase. In a system that contains 100 times more water than WIOM (Figure 6b), even cyclohexene-derived dimers with two H-bond donors partition to the aqueous phase in large fractions (up to 100%). However, there are some cyclohexene-derived dimers that remain in the WIOM phase even at high water content. Prisle et al. found the same as expressed by a very low experimentally derived hydrophilicity factor for α-pinene + O_3_ SOA at high RH.^60^

Recently, Lumiaro et al.^72^ used a machine learning-based method to predict COSMO*therm* estimated saturation vapor pressures and gas-to-particle partitioning coefficients. Such methods may be used to replace computationally heavy COSMO*therm* calculations in the future. However, a large training set of more accurate COSMO*therm* predictions and more complex molecules is needed before COSMO*therm* calculations can be fully replaced by such methods.

## Supporting Information Available

The Supporting Information is available free of charge at https://pubs.acs.org/doi/10.1021/acs.jpca.0c11328. Cosmo and energy files of all studied dimers have been deposited in a reliable public data repository (the CERN Zenodo service) and can be accessed at: https://doi.org/10.5281/zenodo.4291305.

Effect of functional groups on COSMO*therm*-estimated properties, formation of dimers, conformers for COSMO*therm* calculations, effect of parametrization and conformers on saturation vapor pressures, chemical structures of the studied dimers, saturation vapor pressures, activity coefficients, partitioning and *C** comparison (PDF)
