# Supplementary material for: Gas-to-Particle Partitioning of Cyclohexene- and α-Pinene-Derived Highly Oxygenated Dimers Evaluated Using COSMOtherm
Source: J Phys Chem A. 2021 Apr 22;125(17):3726–38. doi: 10.1021/acs.jpca.0c11328 (PMC8154597; doi:10.1021/acs.jpca.0c11328)
Supplement: Supplementary file 1 — jp0c11328_si_001.pdf [file jp0c11328_si_001.pdf]

Supporting Information: Gas-to-Particle Partitioning of Cyclohexene- and α-Pinene-Derived Highly Oxygenated Dimers Evaluated Using COSMOtherm

Noora Hyttinen,[*,†,‡] Matthieu Wolf,[¶] Matti P. Rissanen,[§] Mikael Ehn,[‖] Otso Peräkylä,[‖] Theo Kurtén,[*,¶] and Nønne L. Prisle[*,†,⊥]

†*Nano and Molecular Systems Research Unit, University of Oulu, 90014 Oulu, Finland*

‡*Department of Applied Physics, University of Eastern Finland, 70211 Kuopio, Finland*

¶*Department of Chemistry and Institute for Atmospheric and Earth System Research (INAR), University of Helsinki, 00014 Helsinki, Finland*

§*Aerosol Physics Laboratory, Physics Unit, Tampere University, 33720 Tampere, Finland*

‖*Institute for Atmospheric and Earth System Research (INAR) / Physics, University of Helsinki, 00014 Helsinki, Finland*

⊥*Center for Atmospheric Research, University of Oulu, 90014 Oulu, Finland*

E-mail: noora.hyttinen@uef.fi; theo.kurten@helsinki.fi; nonne.prisle@oulu.fi

S1 The Effect of Functional Groups on COSMO*therm*-Estimated Properties

In COSMO*therm*, the interaction between molecules is described using screening charge densities of the molecules. The screening charge densities are represented as σ-surfaces, which are screening charge surfaces of small surface segments summed over the whole molecule surface. A distribution function of the σ-profile, called a σ-profile, gives the relative amount of each charge surface in the compound (conformer). Increasing the number of H-bond donors increases the positive partial charge surrounding the molecule, when the number of intramolecular H-bonds is limited. This can be seen in Figure S1, where we have plotted the σ-surfaces of the lowest-energy conformers of five different α-pinene-derived dimers containing no intramolecular H-bonds. There is a clear increase in the σ-profile at the $-0.02 < \sigma < -0.01$ e Å^{-2} range when the number of H-bond donors increases (negative σ values correspond to positive partial charges). More extreme partial charges (positive or negative) lead to stronger interaction between molecules, which creates a more stable condensed phase, leading to lower saturation vapor pressures. On the other hand, limiting the number of intramolecular H-bonds in the gas phase leads to less favorable gas-phase energies. Limiting the number of intramolecular H-bonds in COSMO*therm* calculations therefore decreases the stability of the compound in the gas phase, leading to lower saturation vapor pressures.

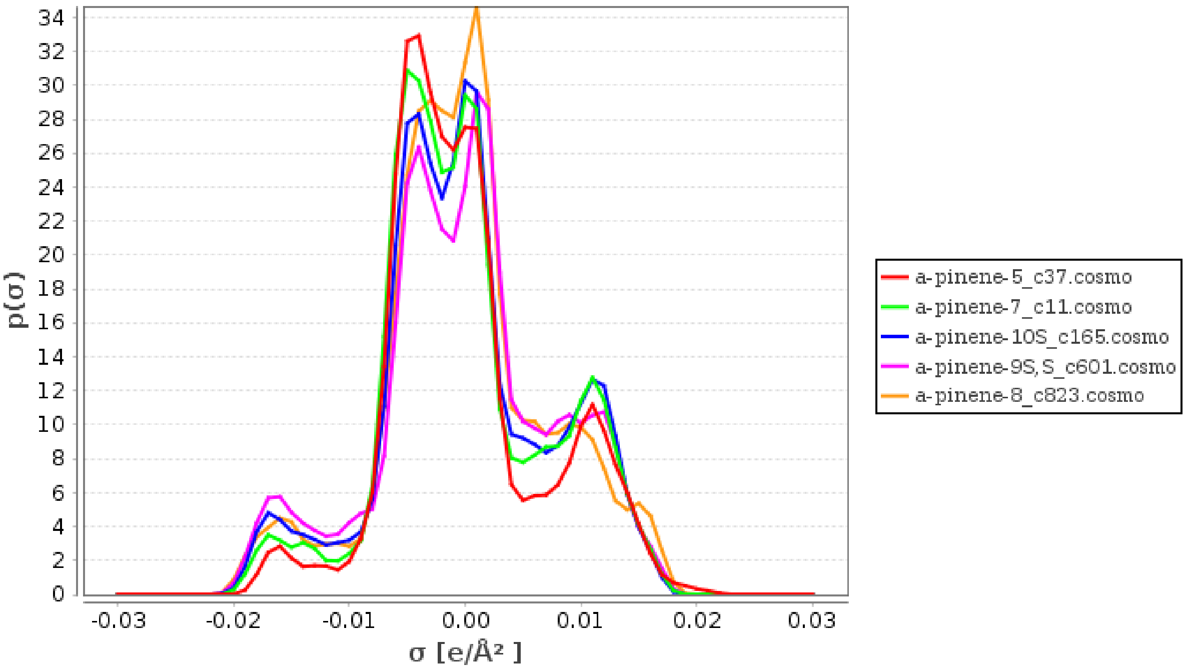

Figure S1: The σ-profiles of lowest-energy conformers containing no intramolecular H-bonds of α-pinene-derived dimers containing 3 (α-pinene-5), 4 (α-pinene-7), 5 (α-pinene-10S and α-pinene-8) and 6 (α-pinene-9S,S) H-bond donors.

The saturation vapor pressure of α-pinene-8 is more than an order of magnitude lower than saturation vapor pressures of α-pinene-10 (both diastereomers, see Figure S9). The opposite difference is seen in the aqueous activity coefficients, where α-pinene-8 has a has an order of magnitude higher activity coefficient than α-pinene-10 (see Figure S13d). The difference between property estimates of the different structural isomers can be a result of multiple differences in the structures of these isomers. Comparing the σ-surfaces of α-pinene-8 and other α-pinene-derived dimers (Figure S1), α-pinene-8 has higher charge density at the positive end of the probability function, caused by the 2 hydroxy groups on the opposite ends of its remaining six-membered ring. Additionally, in contrast to the other studied α-pinene-derived dimers, α-pinene-8 contains a peroxy acid group and has only one ring structure.

Similarly, α-pinene-1 and α-pinene-3 have two six-membered rings and an additional four-membered carbon ring. The saturation vapor pressures of these two dimers are higher

relative to other dimers that contain the same number of H-bond donors and intramolecular H-bonds but no four-membered carbon rings. This indicates that ring structures in HOMs lead to higher saturation vapor pressures, likely due to reduced intermolecular interaction in the condensed phase. However, here the difference in the activity coefficients of these two dimers compared to the other α-pinene-derived dimers is not as large as with α-pinene-8, indicating that the other differences between α-pinene-8 and α-pinene-10 (hydroxide and peroxy acid groups) also play a role in the activity coefficient calculations.

There is no significant difference in the COSMO*therm*-estimated properties of the studied cyclohexene-derived dimers derived from different dimerization reactions, apart from the relatively high vapor pressure of the trioxide dimer, cyclohexene-O10-13. The high saturation vapor pressure is likely due to the relatively low gas-phase energy, rather than high condensed-phase energy. In the conformers that contain no intramolecular H-bonds, one of the trioxide O-O bonds is elongated, indicating that, at the used level of theory (BP/def-TZVP), the trioxide group may not be stable in the gas phase. This may explain the large difference in saturation vapor pressures while the activity coefficient of cyclohexene-O10-13 is similar to the other cyclohexene-derived dimers containing 2 H-bond donors and 1 intramolecular H-bond.

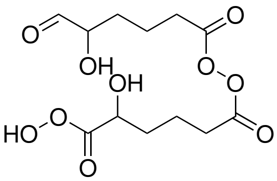
cyclohexene-O10-1

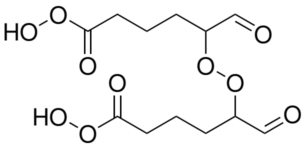
cyclohexene-O10-2

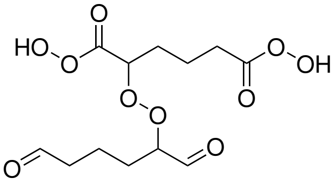
cyclohexene-O10-3

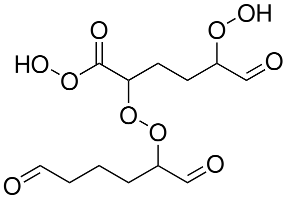
cyclohexene-O10-4

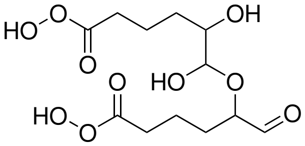
cyclohexene-O10-5

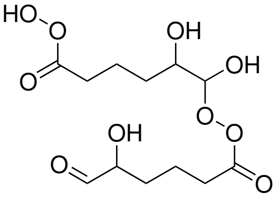
cyclohexene-O10-6

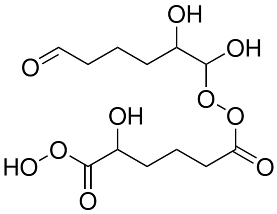
cyclohexene-O10-7

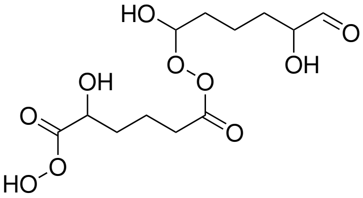
cyclohexene-O10-8

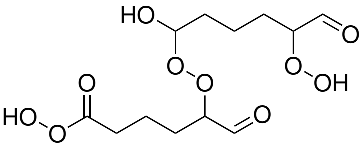
cyclohexene-O10-9

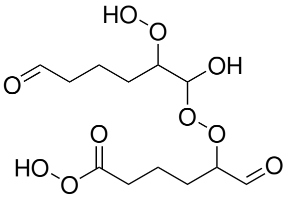
cyclohexene-O10-10

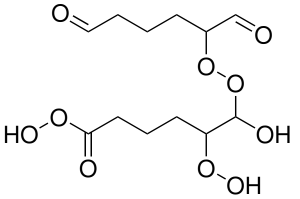
cyclohexene-O10-11

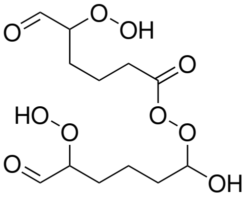
cyclohexene-O10-12

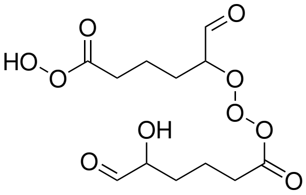
cyclohexene-O10-13

Figure S2: Structures of the studied cyclohexene-derived dimers containing 10 oxygen atoms. We selected the R diastereomers (all stereocenters) for all studied cyclohexene-derived dimers, apart from the ones that are individually named in the main text, and cyclohexene-O10-8, which is the S diastereomer. Color coding: blue - reaction 1, red - reaction 2, magenta - reaction 3, black - reaction 4.

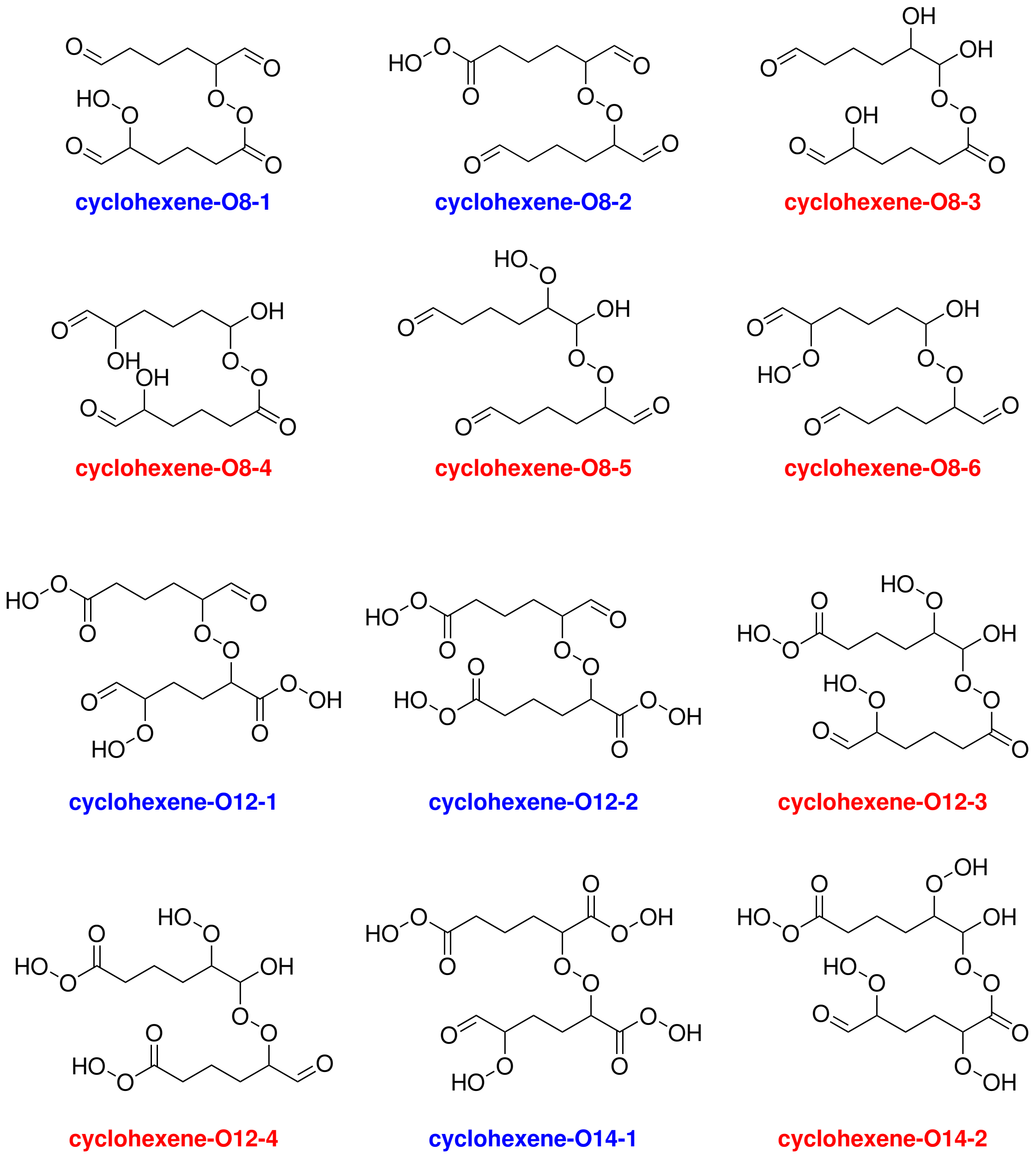

Figure S3: Structures of the studied cyclohexene-derived dimers containing 8, 12 or 14 oxygen atoms. Only R diastereomers of the dimers containing chiral centers were used in this study. Color coding: blue - reaction 1, red - reaction 2.

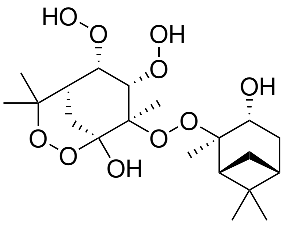

α-pinene-1

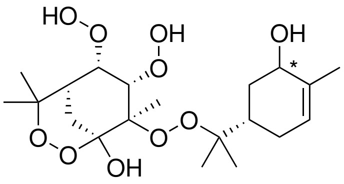

α-pinene-2*

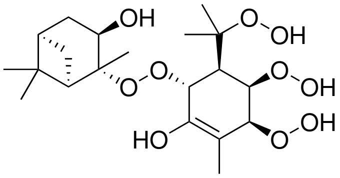

α-pinene-3

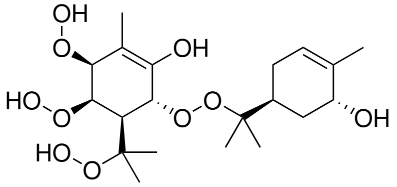

α-pinene-4

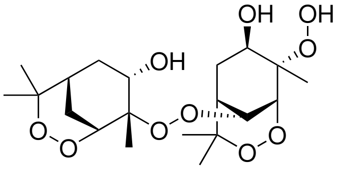

α-pinene-5

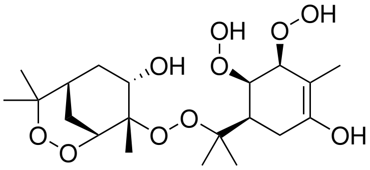

α-pinene-6

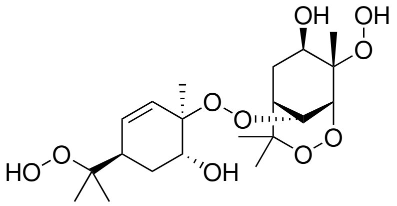

α-pinene-7

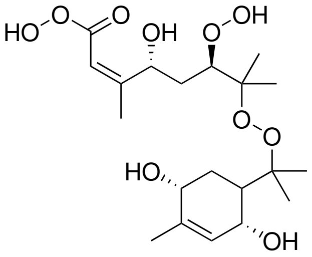

α-pinene-8

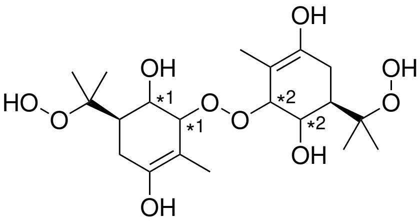

α-pinene-9^{*1,*2}

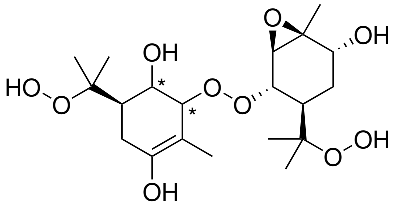

α-pinene-10*

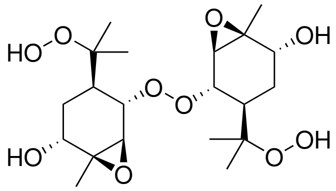

α-pinene-11

Figure S4: Structures of the studied α-pinene-derived dimers. The chiral centers marked with stars are used in the naming of the different diastereomers that were considered.

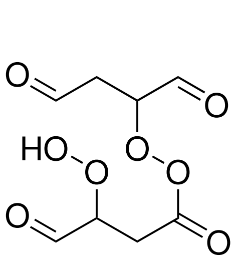

cyclobutene-O8-1

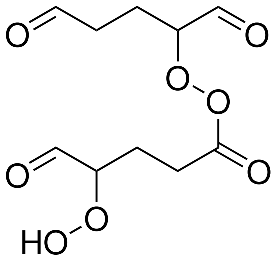

cyclopentene-O8-1

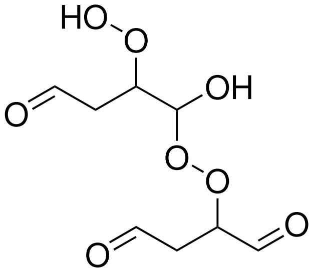

cyclobutene-O8-5

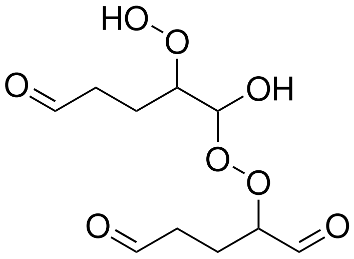

cyclopentene-O8-5

Figure S5: Structures of the studied cyclobutene- and cyclopentene-derived dimers containing 8 oxygen atoms. All isomers are R diastereomers. Color coding: blue - reaction 1, red - reaction 2.

S2 Formation of Dimers

Rissanen et al.[S1] studied the autoxidation of cyclohexene. The cyclohexene-derived dimers studied here are formed from autoxidation products derived by Rissanen et al., with corrections from the formation of alkoxy radicals (from reactions with NO[S2]) and rapid H-shift reactions between alkoxy, peroxy and peroxy acid groups (i.e., "scrambling").[S3–S5] For example, the monomers of cyclohexene-O10-1 are formed when first, the peroxy radicals react with NO to form an alkoxy radical:[S6]

$$\mathrm{ROO^{\bullet} + NO \rightarrow RO^{\bullet} + NO_2} \tag{1}$$

after which an intramolecular H-shift in the product alkoxy radical leads to a hydroxide peroxy radical:[S7]

$$\mathrm{HOORO^{\bullet} \rightarrow HOROO^{\bullet}} \tag{2}$$

Both RO_2 and $HORO_2$ radicals can recombine to form dimers:

$$\mathrm{ROO^{\bullet} + R'OO^{\bullet} \rightarrow ROOR' + O_2} \tag{3}$$

For the trioxide (cyclohexene-O10-13) formation, an alkoxy radical from reaction 1 has reacted with a peroxy radical.

For closed-shell reactions, hydroperoxides are formed in RO_2 radical reaction with HO_2 radicals:[S8]

$$\mathrm{ROO^{\bullet} + HO_2 \rightarrow ROOH + O_2} \tag{4}$$

These hydroperoxides (and hydroxides) form dimers by reacting with the aldehyde groups of other closed shell products in the condensed phase reactions:

$$\mathrm{ROOH + R'{=}O \rightarrow ROOR'OH} \tag{5}$$

$$\mathrm{ROH} + \mathrm{R'{=}O} \rightarrow \mathrm{ROR'OH} \tag{6}$$

The studied α-pinene-derived dimers were formed from monomer intermediates of α-pinene + OH autoxidation proposed by Berndt et al.[S9] and Xu et al.[S10]

S3 Conformers for COSMO*therm* Calculations

Selecting conformers based on their intramolecular H-bonding has previously been studied in aqueous systems, as well as pure multifunctional compounds.[S11,S12] In this study, we estimate activity coefficients of multifunctional compounds in a water insoluble organic molecule (WIOM). This compound is only able to act as a H-bond acceptor unlike water and multifunctional compounds that contain multiple H-bond donors. Here, we investigate whether the optimal conformer set for highly oxygenated multifunctional compounds in a WIOM solution is the same as in water and the pure compound.

For these calculations, we selected 4 α-pinene-derived dimers, all containing different number of H-bond donors. First, we selected conformers containing different numbers of intramolecular H-bonds. Each of these conformers is the lowest-energy conformer of the compound that was found containing that specific number of H-bonds. We then computed pseudo-chemical potentials of each dimer conformer at infinite dilution in WIOM and water, as well as the pure dimer solution, to obtain the favorability of each conformer in each solution. As the pure dimer solution, we used a set of conformers where each conformer contain a different number of intramolecular H-bonds, each having the lowest energy of all found conformers that contain the same number of intramolecular H-bonds.

Table S1 shows the pseudo-chemical potentials of each conformer in the different solutions. Conformers containing the maximum number of intramolecular H-bonds were higher in energy than some conformers containing fewer H-bonds, and those are not shown in the table. For instance, the lowest-energy conformer of α-pinene-5 containing 3 intramolecular H-bonds is higher in energy than the the lowest-energy conformer containing 2 intramolecular H-bonds, which is the lowest-energy conformer among all found conformers. We see that in each solution, the conformer containing the lowest number of intramolecular H-bonds has the lowest pseudo-chemical potential, with the exception of α-pinene-9S,S containing no intramolecular H-bonds. However, we were not able to find a sufficient number of conformers containing no intramolecular H-bonds for α-pinene-9S,S, and the 1 H-bond conformer set

was therefore used in the COSMO*therm* calculations.

Table S1: Pseudo-chemical potentials (μ^* in kJ mol^{-3}) of the lowest-energy conformers in each conformer set in water, WIOM[S13] and the pure dimer. The conformers with the highest number of intramolecular H-bonds shown here are the lowest-energy conformers in the full set of conformers.

# H-bonds	$\mu^{*,\mathrm{w}}$	$\mu^{*,\mathrm{WIOM}}$	$\mu^{*,\mathrm{pure}}$
α-pinene-5 (3 donors)			
2	10.90	6.35	3.05
1	6.45	7.99	4.11
0	5.52	3.49	1.05
α-pinene-7 (4 donors)			
2	22.63	17.27	9.71
1	20.32	15.46	8.62
0	-4.08	-2.59	-8.49
α-pinene-10S (5 donors)			
3	30.91	26.26	20.92
2	30.10	25.01	19.32
1	6.34	3.32	0.05
0	-0.71	2.70	-2.44
α-pinene-9S,S (6 donors)			
4	30.75	28.21	21.92
3	27.24	23.43	18.28
2	12.97	19.98	11.30
1	5.84	4.90	1.46
0	3.56	13.15	9.37

The change in pseudo-chemical potentials when the conformer set is changed is similar in the pure and the WIOM solutions (see Table S1). In aqueous solutions, the pseudo-chemical potential of the multifunctional dimers changes generally more than in the pure solutions or WIOM, when the number of intramolecular H-bonds is limited. The effect of selecting conformers in COSMO*therm* is therefore smaller when calculating properties in WIOM solutions than in aqueous solutions, using the pure compound reference.

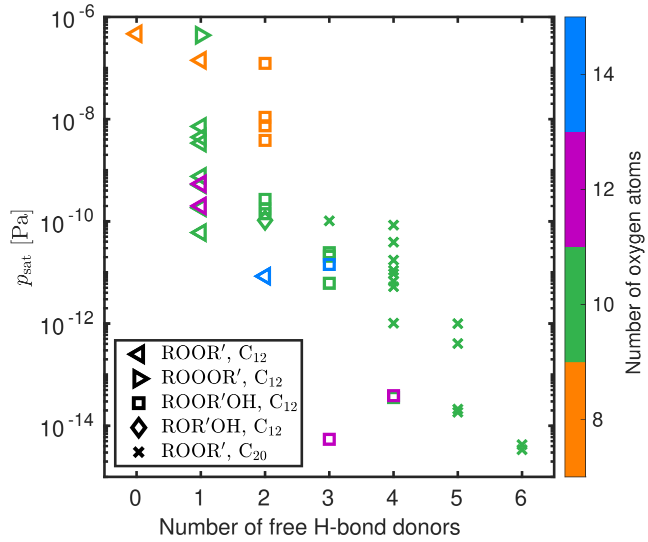

Figure S6: COSMO*therm*-estimated saturation vapor pressures of cyclohexene and α-pinene-derived dimers as a function of number of free H-bond donors in each molecule. Different markers represent different dimer formation reactions (triangles and crosses for gas-phase and quadrangles for condensed-phase reactions), and the number of oxygen atoms is indicated with different colors.

Table S2: COSMO*therm*-estimated activity coefficients (γ) and saturation vapor pressures (p_{sat} in Pa) at 298.15 K. SIMPOL.1-estimated saturation vapor pressures (p_{sat},SIMPOL.1 in Pa) at 298.15 K.

Compound	γ^{w}	γ^{WIOM}	p_{sat}	p_{sat},SIMPOL.1
cyclohexene-O8-1	7.34×10^{2}	4.28	1.42×10^{-7}	1.08×10^{-6}
cyclohexene-O8-2	7.17×10^{3}	2.29	4.68×10^{-7}	9.60×10^{-6}
cyclohexene-O8-3	2.70×10^{1}	1.51×10^{1}	7.40×10^{-9}	1.76×10^{-9}
cyclohexene-O8-4	2.36×10^{1}	1.57×10^{1}	1.08×10^{-8}	1.76×10^{-9}
cyclohexene-O8-5	7.15×10^{1}	8.68	3.84×10^{-9}	6.15×10^{-8}
cyclohexene-O8-6	1.91×10^{2}	5.96	1.23×10^{-7}	6.15×10^{-8}
cyclohexene-O10-1R,S	2.70×10^{3}	3.19	4.44×10^{-9}	2.41×10^{-9}
cyclohexene-O10-1S,S	8.21×10^{2}	5.20	7.20×10^{-9}	2.41×10^{-9}
cyclohexene-O10-2R,S	2.02×10^{3}	3.22	6.03×10^{-11}	7.46×10^{-7}
cyclohexene-O10-2S,S	5.67×10^{3}	2.76	5.20×10^{-10}	7.46×10^{-7}
cyclohexene-O10-3R,S	1.62×10^{2}	7.35	1.85×10^{-10}	7.46×10^{-7}
cyclohexene-O10-3S,S	5.95×10^{3}	2.31	7.58×10^{-10}	7.46×10^{-7}
cyclohexene-O10-4	9.13×10^{2}	4.81	3.40×10^{-9}	3.49×10^{-8}
cyclohexene-O10-5	2.33×10^{2}	3.74	1.05×10^{-10}	3.28×10^{-10}
cyclohexene-O10-6	3.95×10^{1}	2.08×10^{1}	6.18×10^{-12}	1.37×10^{-10}
cyclohexene-O10-7	2.44×10^{1}	1.72×10^{1}	2.09×10^{-11}	1.37×10^{-10}
cyclohexene-O10-8	5.40	2.63×10^{1}	3.57×10^{-14}	1.37×10^{-10}
cyclohexene-O10-9	7.56×10^{2}	3.60	1.76×10^{-10}	4.78×10^{-9}
cyclohexene-O10-10	4.01×10^{2}	4.94	2.70×10^{-10}	4.78×10^{-9}
cyclohexene-O10-11	5.00×10^{2}	4.97	1.41×10^{-10}	4.78×10^{-9}
cyclohexene-O10-12	7.84×10^{1}	9.12	2.42×10^{-11}	5.40×10^{-10}
cyclohexene-O10-13	2.52×10^{3}	1.59	4.38×10^{-7}	-
cyclohexene-O12-1	2.26×10^{3}	9.73	5.42×10^{-10}	2.72×10^{-9}
cyclohexene-O12-2	3.01×10^{4}	1.31	2.00×10^{-10}	5.81×10^{-8}
cyclohexene-O12-3	3.08×10^{1}	1.92×10^{1}	5.45×10^{-15}	4.20×10^{-11}
cyclohexene-O12-4	6.56	1.18×10^{1}	3.89×10^{-14}	3.72×10^{-10}
cyclohexene-O14-1	5.93×10^{2}	1.66	8.45×10^{-12}	2.11×10^{-10}
cyclohexene-O14-2	3.27×10^{1}	3.09	1.44×10^{-11}	1.53×10^{-13}
cyclobutene-O8-1	9.24×10^{1}	6.72	1.05×10^{-5}	5.38×10^{-5}
cyclobutene-O8-5	7.80	1.81×10^{1}	5.90×10^{-7}	3.06×10^{-6}
cyclopentene-O8-1	2.70×10^{2}	4.85	9.44×10^{-7}	7.63×10^{-6}
cyclopentene-O8-5	1.64×10^{1}	1.25×10^{1}	1.33×10^{-7}	4.34×10^{-7}

Table S3: COSMO*therm*-estimated activity coefficients (γ) and saturation vapor pressures (p_{sat} in Pa) at 298.15 K. SIMPOL.1-estimated saturation vapor pressures (p_{sat},SIMPOL.1 in Pa) at 298.15 K.

Compound	γ^{w}	γ^{WIOM}	p_{sat}	p_{sat},SIMPOL.1
α-pinene-1	4.94	4.24	8.45×10^{-11}	1.77×10^{-12}
α-pinene-2R	1.01×10^{1}	6.30	3.92×10^{-11}	1.45×10^{-12}
α-pinene-2S	1.48×10^{1}	6.84	1.74×10^{-11}	1.45×10^{-12}
α-pinene-3	2.73×10^{1}	6.34	9.44×10^{-12}	1.31×10^{-14}
α-pinene-4	3.87×10^{1}	6.74	1.02×10^{-12}	1.07×10^{-14}
α-pinene-5	4.01	1.84	1.02×10^{-10}	1.96×10^{-10}
α-pinene-6	5.46	1.05×10^{1}	1.10×10^{-11}	1.45×10^{-12}
α-pinene-7	7.77	1.22×10^{1}	5.27×10^{-12}	1.45×10^{-12}
α-pinene-8	5.04×10^{1}	1.05×10^{1}	2.11×10^{-14}	2.09×10^{-14}
α-pinene-9R,R	1.86×10^{-1}	8.22	3.41×10^{-15}	1.27×10^{-16}
α-pinene-9S,R	4.93×10^{-1}	1.10×10^{1}	4.27×10^{-15}	1.27×10^{-16}
α-pinene-9S,S	3.18	6.81	1.85×10^{-14}	1.27×10^{-16}
α-pinene-10R	2.17	5.42	9.97×10^{-13}	5.09×10^{-15}
α-pinene-10S	2.02	6.44	4.07×10^{-13}	5.09×10^{-15}
α-pinene-11	2.79×10^{1}	4.08	6.74×10^{-12}	2.03×10^{-13}

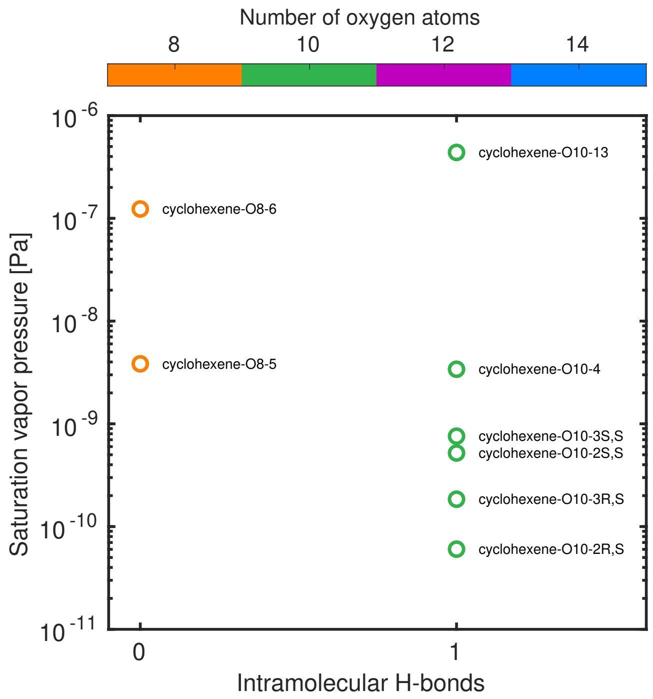

Figure S7: COSMO*therm*-estimated saturation vapor pressures of dimers containing 2 H-bond donors as a function of the number of intramolecular H-bonds in the conformer set used in the COSMO*therm* calculation.

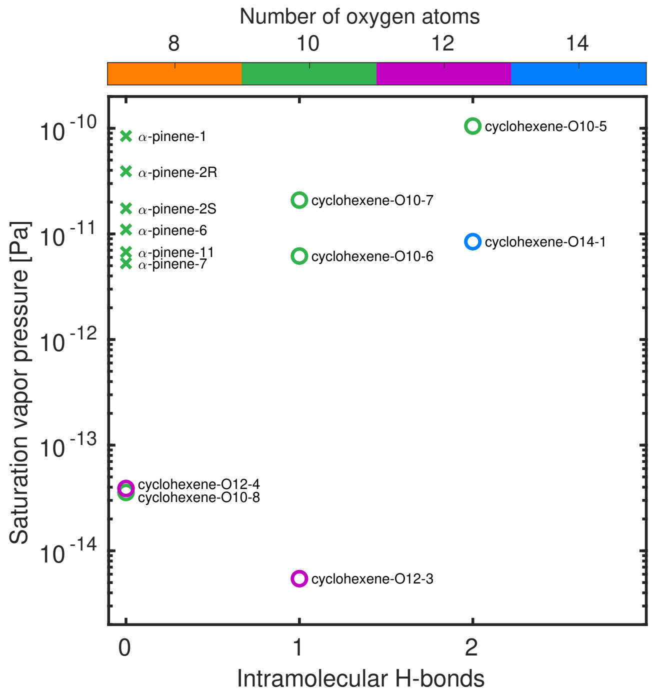

Figure S8: COSMO*therm*-estimated saturation vapor pressures of dimers containing 4 H-bond donors as a function of the number of intramolecular H-bonds in the conformer set used in the COSMO*therm* calculation.

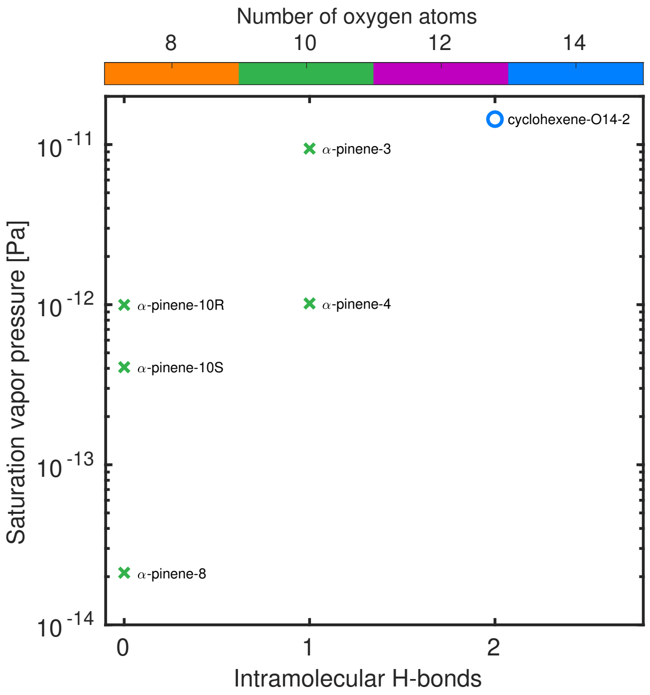

Figure S9: COSMO*therm*-estimated saturation vapor pressures of dimers containing 5 H-bond donors as a function of the number of intramolecular H-bonds in the conformer set used in the COSMO*therm* calculation.

S4 The Effect of Parametrization and Conformers on Saturation Vapor Pressures

We recomputed saturation vapor pressures of two HOM monomers and two HOM dimers derived from α-pinene ozonolysis investigated by Kurtén et al.[S14] The selected monomers contain three H-bond donors each, while the dimers contain one or two H-bond donors (see Figure S10).

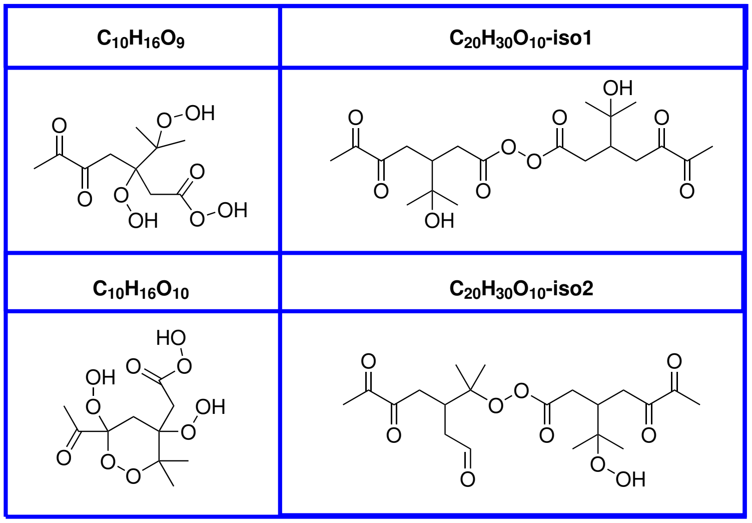

Figure S10: Selected HOM monomer ($C_{10}H_{16}O_9$ and $C_{10}H_{16}O_{10}$) and dimer ($C_{20}H_{30}O_{10}$-iso1 and $C_{20}H_{30}O_{10}$-iso2) structures from Kurtén et al.[S14]

We found conformers that contain no intramolecular H-bonds using the systematic conformer sampling method.[S11] Saturation vapor pressures were calculated using these conformers, as well as the conformers used by Kurtén et al., using both BP_TZVPD_FINE_C30_1501 and BP_TZVPD_FINE_19 parametrizations. These saturation vapor pressures are compared with those estimated using the SIMPOL.1 group-contribution method[S15] in Figure S11. It should be noted that Kurtén et al. computed only a single conformer of the dimer HOMs ($C_{20}H_{30}O_{10}$-iso1 and $C_{20}H_{30}O_{10}$-iso2) at the BP/def2-TZVPD-FINE level of theory, which leads to additional uncertainties in COSMO*therm* calculations.

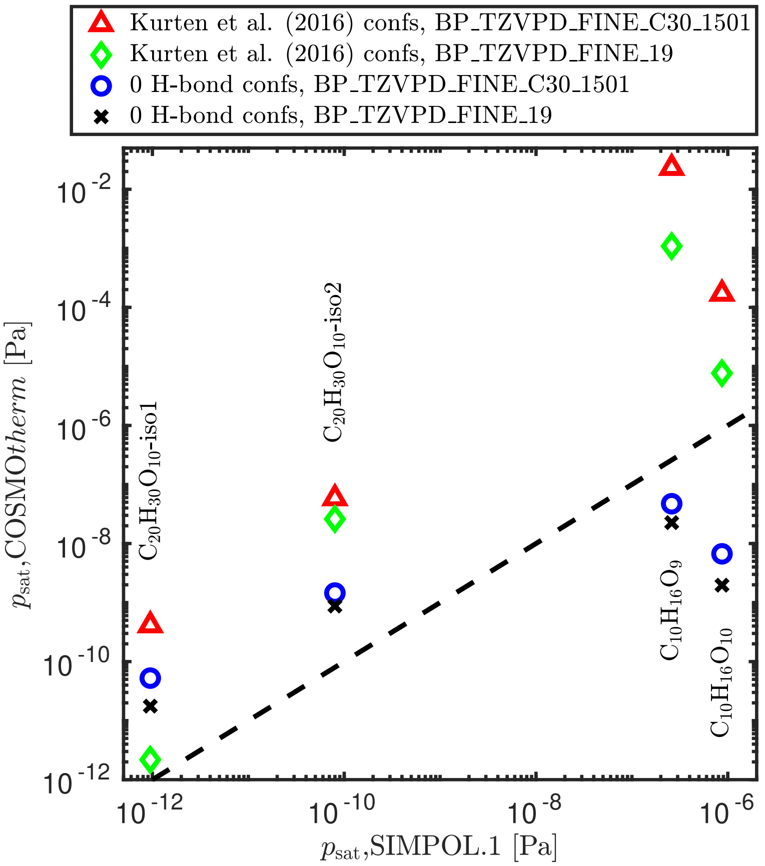

Figure S11: Comparison between SIMPOL.1- and COSMO*therm*-estimated saturation vapor pressures of α-pinene-derived HOM monomers and dimers from Kurtén et al.[S14] at 298.15 K. Conformers used in COSMO*therm* calculations were either taken from Kurtén et al.[S14] (triangles and diamonds) or found using a systematic conformer sampling method and excluding conformers that contain intramolecular H-bonds (circles and crosses). All calculations were run using COSMO*therm*19 and either BP_TZVPD_FINE_C30_1501 (triangles and circles) or BP_TZVPD_FINE_19 (diamonds and crosses) parametrization. The dashed line shows 1:1 ratio between COSMO*therm* and SIMPOL.1 estimates.

The difference between the two used parametrizations is larger when conformers containing intramolecular H-bonds are used in the COSMO*therm* calculation (HOM monomers). With our systematic conformer sampling and conformer selection, only little difference is seen between the parametrizations. The largest effect on the saturation vapor pressures is seen with the conformers selected for the COSMO*therm* calculations. For the HOM monomers (three H-bond donors), the saturation vapor pressures estimated using conformers containing multiple intramolecular H-bonds are several orders of magnitude (3-5) higher

than those estimated using conformers that contain no intramolecular H-bonds. In addition, COSMO*therm* predicts saturation vapor pressures that are lower than those predicted by SIMPOL.1. For the HOM dimers (two and one H-bond donors), the Kurtén et al.[S14] used a single conformer, both containing one intramolecular H-bond. SIMPOL.1 predicts lower p_{sat} than COSMO*therm* for the C_{20} dimers, while for the C_{10} and C_{12} HOMs, COSMO*therm* predicts lower p_{sat} than SIMPOL.1.

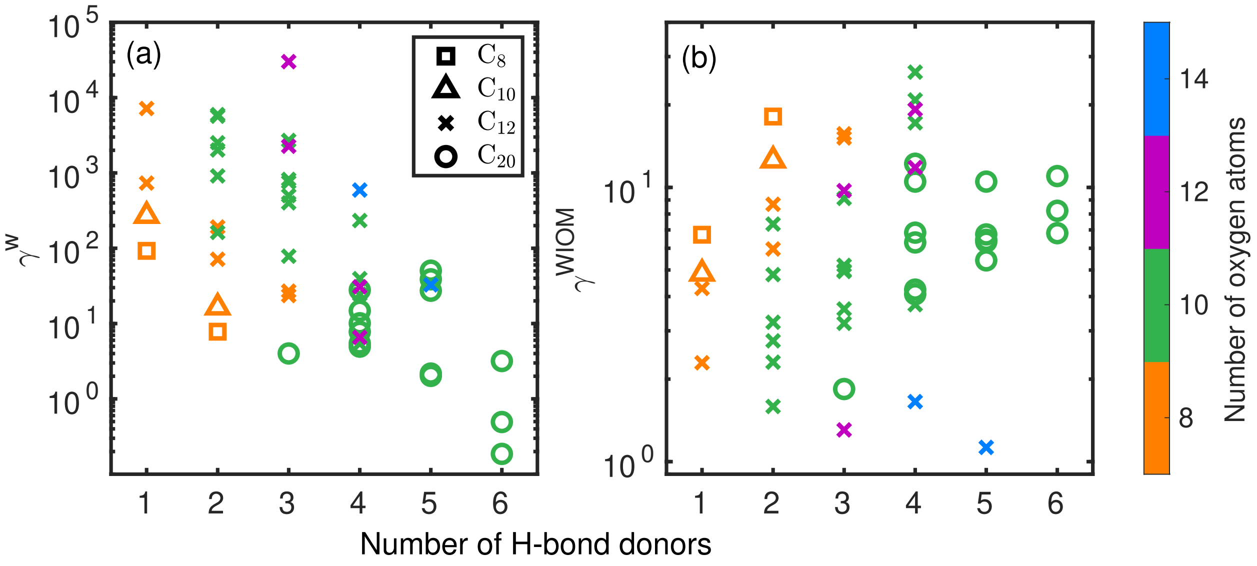

Figure S12: COSMO*therm*-estimated activity coefficients of the studied dimers at infinite dilution in (a) water and (b) WIOM as a function of number of free H-bond donors. Different markers represent different conformer sets used in COSMO*therm* calculations, and the number of oxygen atoms is shown with different colors.

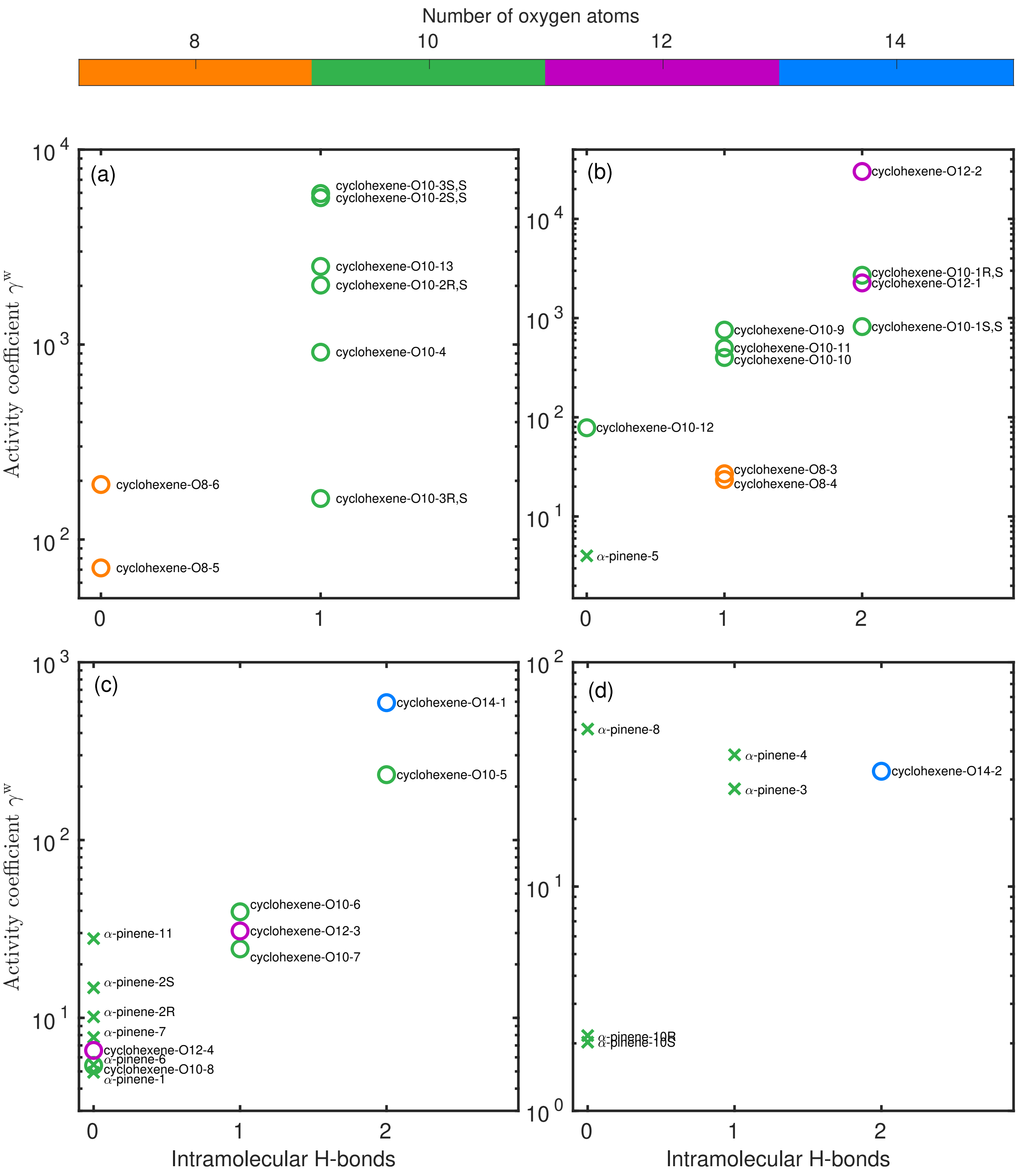

Figure S13: COSMO*therm*-estimated aqueous activity coefficients (γ^{w}) of dimers containing (a) 2, (b) 3, (c) 4 and (d) 5 H-bond donors at 298.15 K.

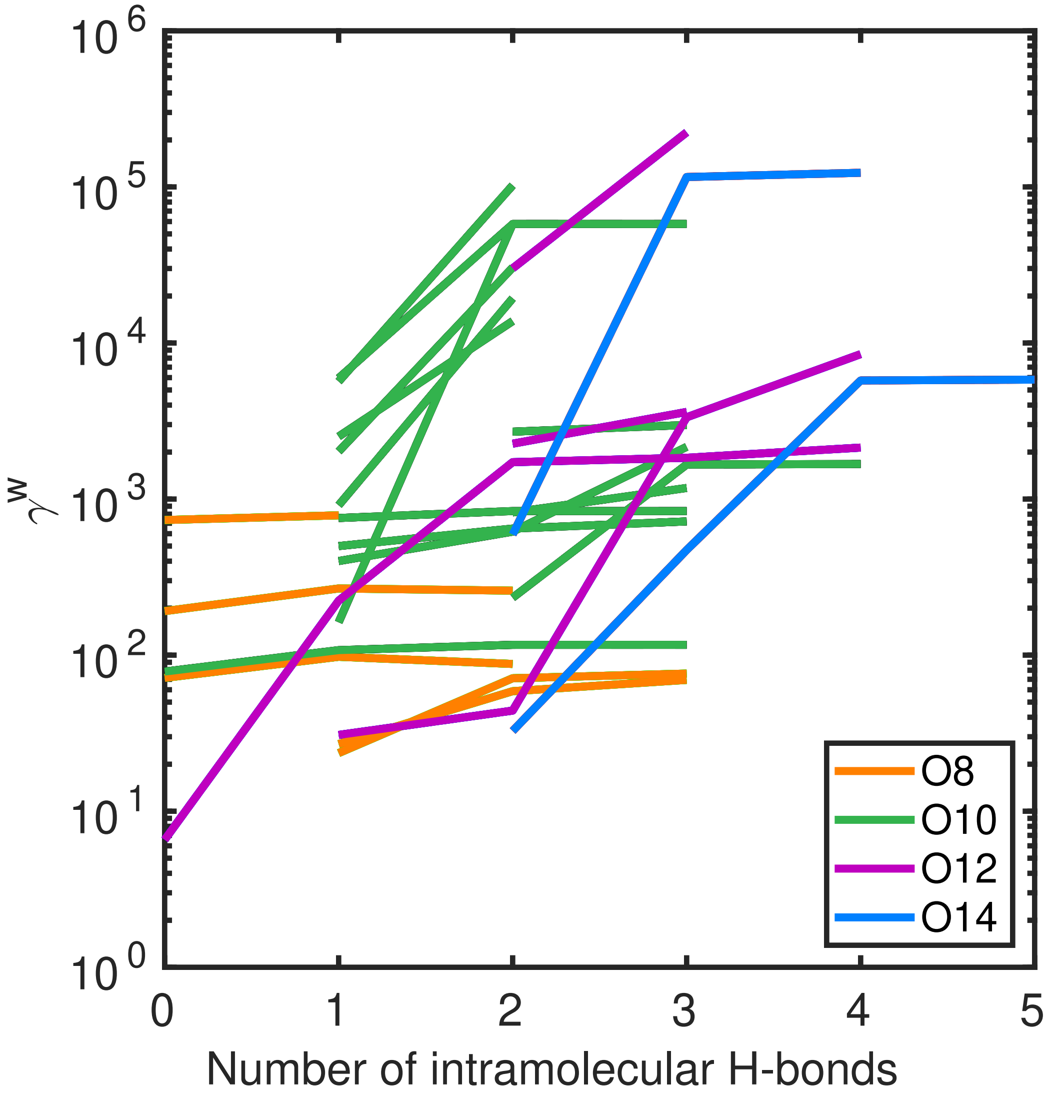

Figure S14: Effect of intramolecular H-bonds on aqueous activity coefficients of studied cyclohexene-derived dimers in COSMO*therm*.

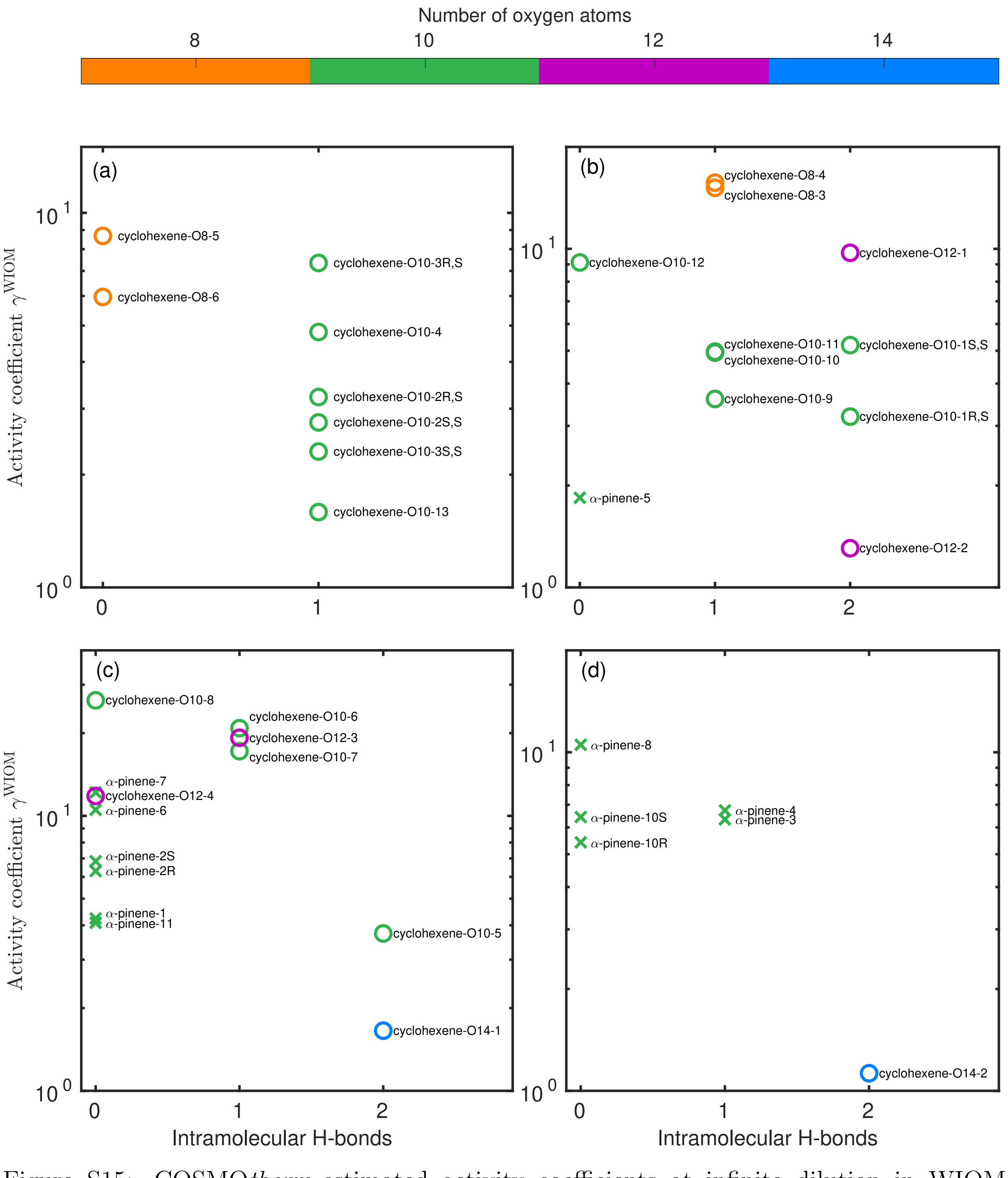

Figure S15: COSMO*therm*-estimated activity coefficients at infinite dilution in WIOM (γ^{WIOM}) of dimers containing (a) 2, (b) 3, (c) 4 and (d) 5 H-bond donors at 298.15 K.

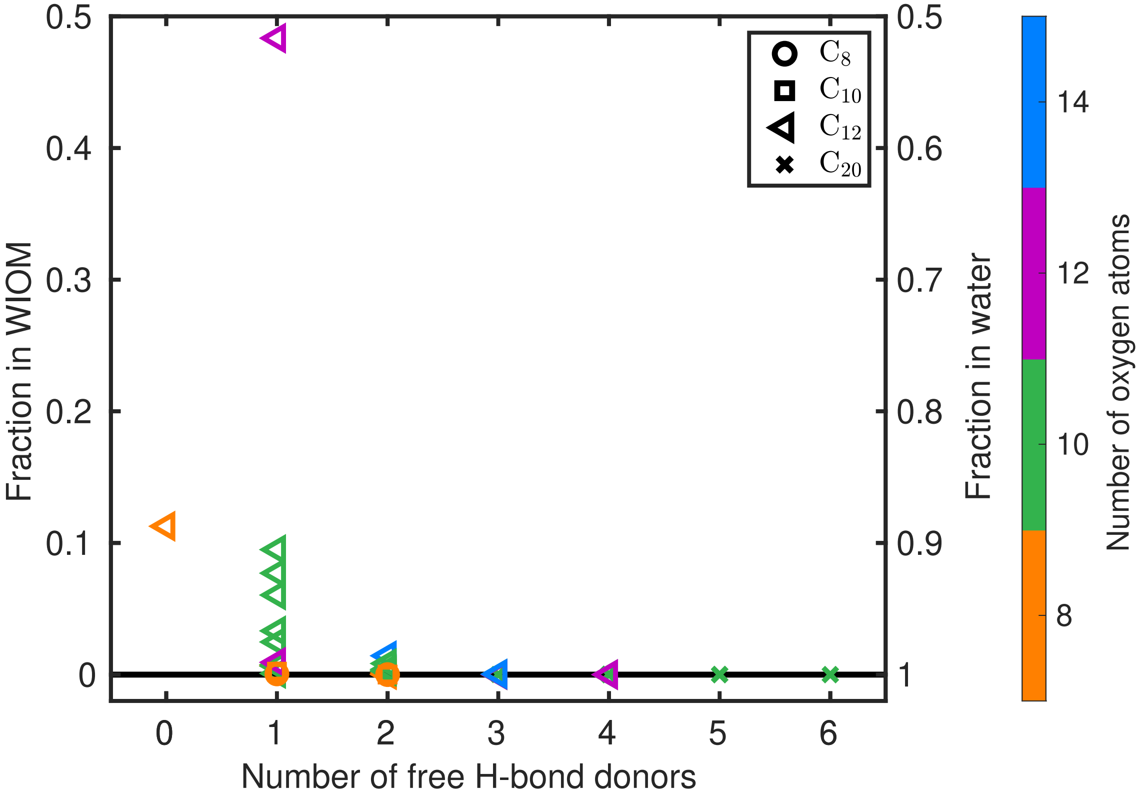

Figure S16: Fractions of dimers in WIOM (left y-axis) and water (right y-axis) in a system that contains 1:30 000 ratio of WIOM and water (cloud scenario in Wang et al.[S16]).

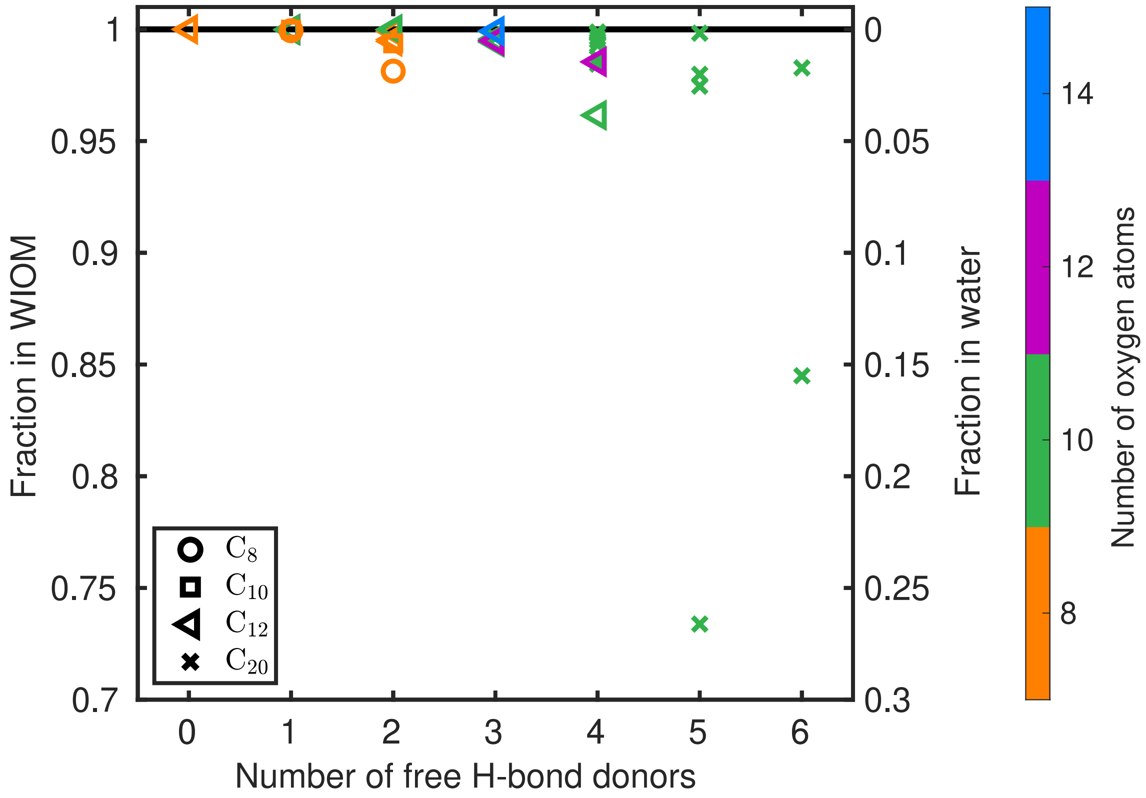

Figure S17: Fractions of dimers in WIOM (left y-axis) and water (right y-axis) in a system that contains 1:0.01 ratio of WIOM and water.

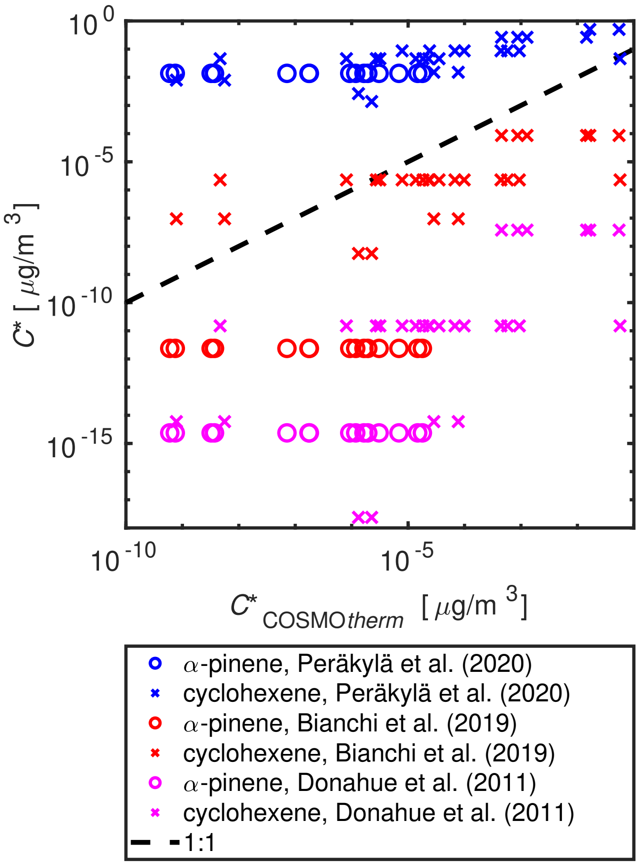

Figure S18: Comparison of saturation mass concentrations (C^* in μg m^{-3}) derived from COSMO*therm*-estimated saturation vapor pressure and empirical models by Donahue et al.,[S17] Bianchi et al.[S18] and Peräkylä et al.[S19]

References

(S1) Rissanen, M. P.; Kurtén, T.; Sipilä, M.; Thornton, J. A.; Kangasluoma, J.; Sarnela, N.; Junninen, H.; Jørgensen, S.; Schallhart, S.; Kajos, M. K.; Taipale, R.; Springer, M.; Mentel, T. F.; Ruuskanen, T.; Petäjä, T.; Worsnop, D. R.; Kjaergaard, H. G.; Ehn, M. The formation of highly oxidized multifunctional products in the ozonolysis of cyclohexene. *J. Am. Chem. Soc.* **2014**, *136*, 15596–15606.

(S2) Mentel, T. F.; Springer, M.; Ehn, M.; Kleist, E.; Pullinen, I.; Kurtén, T.; Rissanen, M.; Wahner, A.; Wildt, J. Formation of highly oxidized multifunctional compounds: autoxidation of peroxy radicals formed in the ozonolysis of alkenes – deduced from structure–product relationships. *Atmos. Chem. Phys.* **2015**, *15*, 6745–6765.

(S3) Jørgensen, S.; Knap, H. C.; Otkjær, R. V.; Jensen, A. M.; Kjeldsen, M. L. H.; Wennberg, P. O.; Kjaergaard, H. G. Rapid hydrogen shift scrambling in hydroperoxy-substituted organic peroxy radicals. *J. Phys. Chem. A* **2016**, *120*, 266–275.

(S4) Knap, H. C.; Jørgensen, S. Rapid hydrogen shift reactions in acyl peroxy radicals. *J. Phys. Chem. A* **2017**, *121*, 1470–1479.

(S5) Hyttinen, N.; Rissanen, M. P.; Kurtén, T. Computational comparison of acetate and nitrate chemical ionization of highly oxidized cyclohexene ozonolysis intermediates and products. *J. Phys. Chem. A* **2017**, *121*, 2172–2179.

(S6) Lohr, L. L.; Barker, J. R.; Shroll, R. M. Modeling the organic nitrate yields in the reaction of alkyl peroxy radicals with nitric oxide. 1. Electronic structure calculations and thermochemistry. *J. Phys. Chem. A* **2003**, *107*, 7429–7433.

(S7) Praske, E.; Otkjær, R. V.; Crounse, J. D.; Hethcox, J. C.; Stoltz, B. M.; Kjaergaard, H. G.; Wennberg, P. O. Intramolecular hydrogen shift chemistry of hydroperoxy-substituted peroxy radicals. *J. Phys. Chem. A* **2018**, *123*, 590–600.

(S8) Glowacki, D. R.; Pilling, M. J. Unimolecular reactions of peroxy radicals in atmospheric chemistry and combustion. *ChemPhysChem* **2010**, *11*, 3836–3843.

(S9) Berndt, T.; Richters, S.; Jokinen, T.; Hyttinen, N.; Kurtén, T.; Otkjær, R. V.; Kjaergaard, H. G.; Stratmann, F.; Herrmann, H.; Sipilä, M.; Kulmala, M.; Ehn, M. Hydroxyl radical-induced formation of highly oxidized organic compounds. *Nat. Commun.* **2016**, *7*, 1–8.

(S10) Xu, L.; Møller, K. H.; Crounse, J. D.; Otkjær, R. V.; Kjaergaard, H. G.; Wennberg, P. O. Unimolecular reactions of peroxy radicals formed in the oxidation of α-pinene and β-pinene by hydroxyl radicals. *J. Phys. Chem. A* **2019**, *123*, 1661–1674.

(S11) Kurtén, T.; Hyttinen, N.; D'Ambro, E. L.; Thornton, J.; Prisle, N. L. Estimating the saturation vapor pressures of isoprene oxidation products $C_5H_{12}O_6$ and $C_5H_{10}O_6$ using COSMO-RS. *Atmos. Chem. Phys.* **2018**, *18*, 17589–17600.

(S12) Hyttinen, N.; Prisle, N. L. Improving Solubility and Activity Estimates of Multifunctional Atmospheric Organics by Selecting Conformers in COSMO*therm*. *J. Phys. Chem. A* **2020**, *124*, 4801–4812.

(S13) Kalberer, M.; Paulsen, D.; Sax, M.; Steinbacher, M.; Dommen, J.; Prévôt, A. S.; Fisseha, R.; Weingartner, E.; Frankevich, V.; Zenobi, R.; Baltensperger, U. Identification of polymers as major components of atmospheric organic aerosols. *Science* **2004**, *303*, 1659–1662.

(S14) Kurtén, T.; Tiusanen, K.; Roldin, P.; Rissanen, M.; Luy, J.-N.; Boy, M.; Ehn, M.; Donahue, N. α-Pinene autoxidation products may not have extremely low saturation vapor pressures despite high O:C ratios. *J. Phys. Chem. A* **2016**, *120*, 2569–2582.

(S15) Pankow, J. F.; Asher, W. E. SIMPOL.1: a simple group contribution method for predicting vapor pressures and enthalpies of vaporization of multifunctional organic compounds. *Atmos. Chem. Phys.* **2008**, *8*, 2773–2796.

(S16) Wang, C.; Yuan, T.; Wood, S. A.; Goss, K.-U.; Li, J.; Ying, Q.; Wania, F. Uncertain Henry's law constants compromise equilibrium partitioning calculations of atmospheric oxidation products. *Atmos. Chem. Phys.* **2017**, *17*, 7529–7540.

(S17) Donahue, N. M.; Epstein, S. A.; Pandis, S. N.; Robinson, A. A two-dimensional volatility basis set: 1. organic-aerosol mixing thermodynamics. *Atmos. Chem. Phys.* **2011**, *11*, 3303–3318.

(S18) Bianchi, F.; Kurtén, T.; Riva, M.; Mohr, C.; Rissanen, M. P.; Roldin, P.; Berndt, T.; Crounse, J. D.; Wennberg, P. O.; Mentel, T. F.; Wildt, J.; Junninen, H.; Jokinen, T.; Kulmala, M.; Worsnop, D. R.; Thornton, J. A.; Donahue, N.; Kjaergaard, H. G.; Ehn, M. Highly oxygenated organic molecules (HOM) from gas-phase autoxidation involving peroxy radicals: A key contributor to atmospheric aerosol. *Chem. Rev.* **2019**, *119*, 3472–3509.

(S19) Peräkylä, O.; Riva, M.; Heikkinen, L.; Quéléver, L.; Roldin, P.; Ehn, M. Experimental investigation into the volatilities of highly oxygenated organic molecules (HOMs). *Atmos. Chem. Phys.* **2020**, *20*, 649–669.
